# Supplementary figures and images for: Two duplicated gsdf homeologs cooperatively regulate male differentiation by inhibiting cyp19a1a transcription in a hexaploid fish
Source: PLoS Genet. 2022 Jun 29;18(6):e1010288. doi: 10.1371/journal.pgen.1010288 (PMC9275722; doi:10.1371/journal.pgen.1010288)

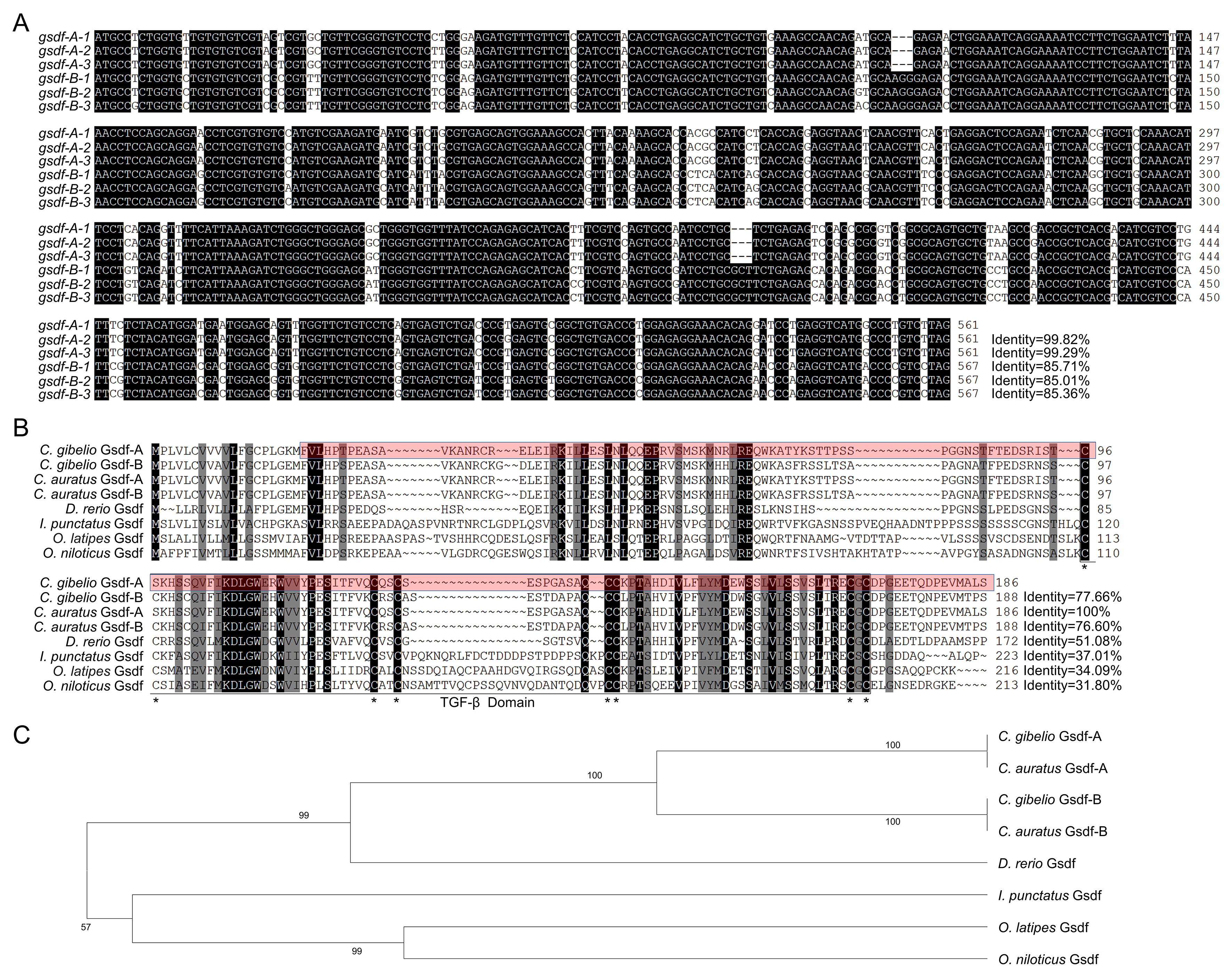

Supplement: S1 Fig — (A) Coding sequence alignment of three gsdf-A alleles and three gsdf-B alleles. Sequence lengths and identities are exhibited at the end of sequences. (B) Multiple amino acid sequence alignment of Gsdf proteins from different fish species. Star marks the conserved cysteine. TGF-β superfamily domain is highlighted by black box. Sequences highlighted by red box were used as antigen for anti-Gsdf antibody preparation. (C) Phylogenetic tree of Gsdf proteins from different fish species. (TIF) [file pgen.1010288.s001.tif]

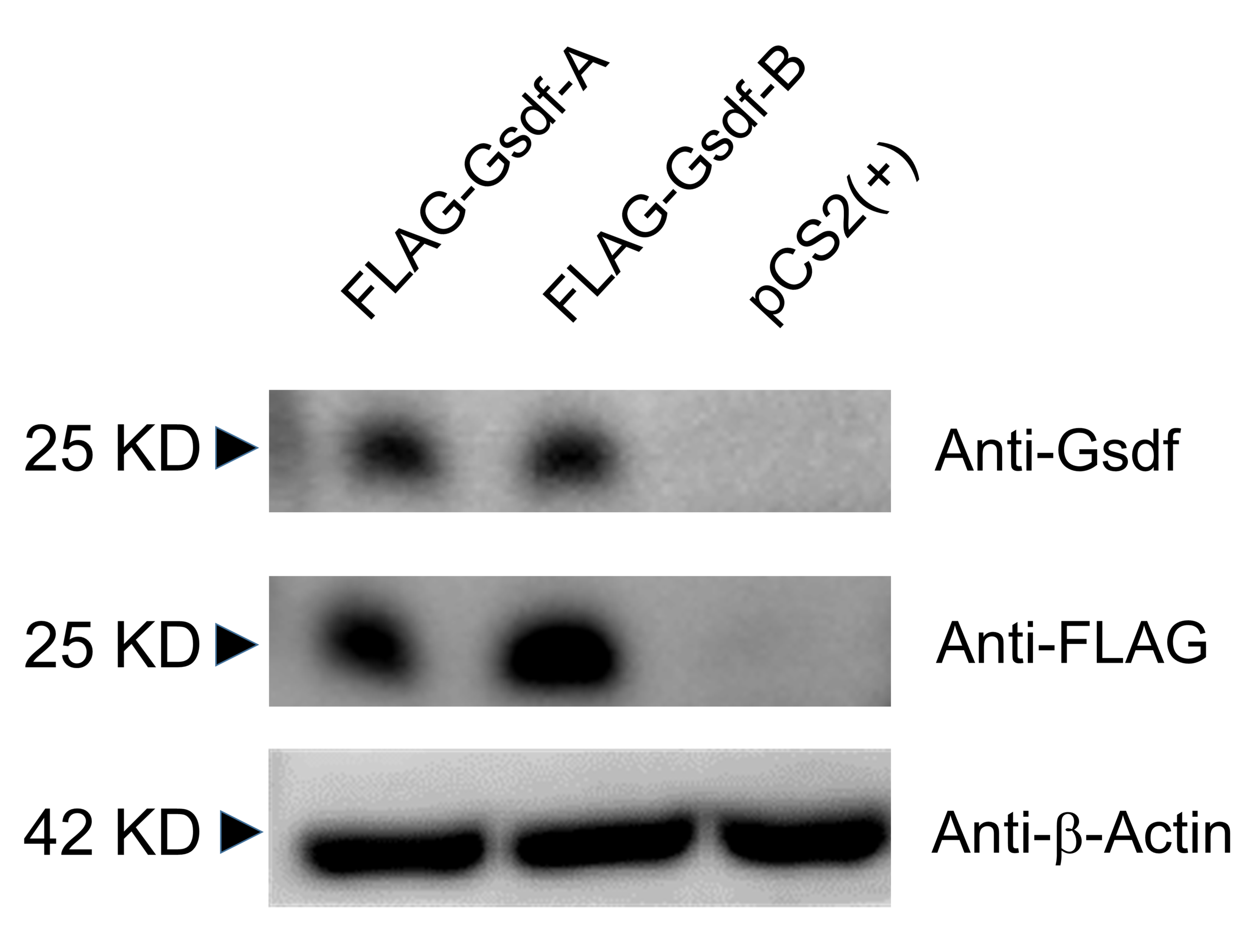

Supplement: S2 Fig — (TIF) [file pgen.1010288.s002.tif]

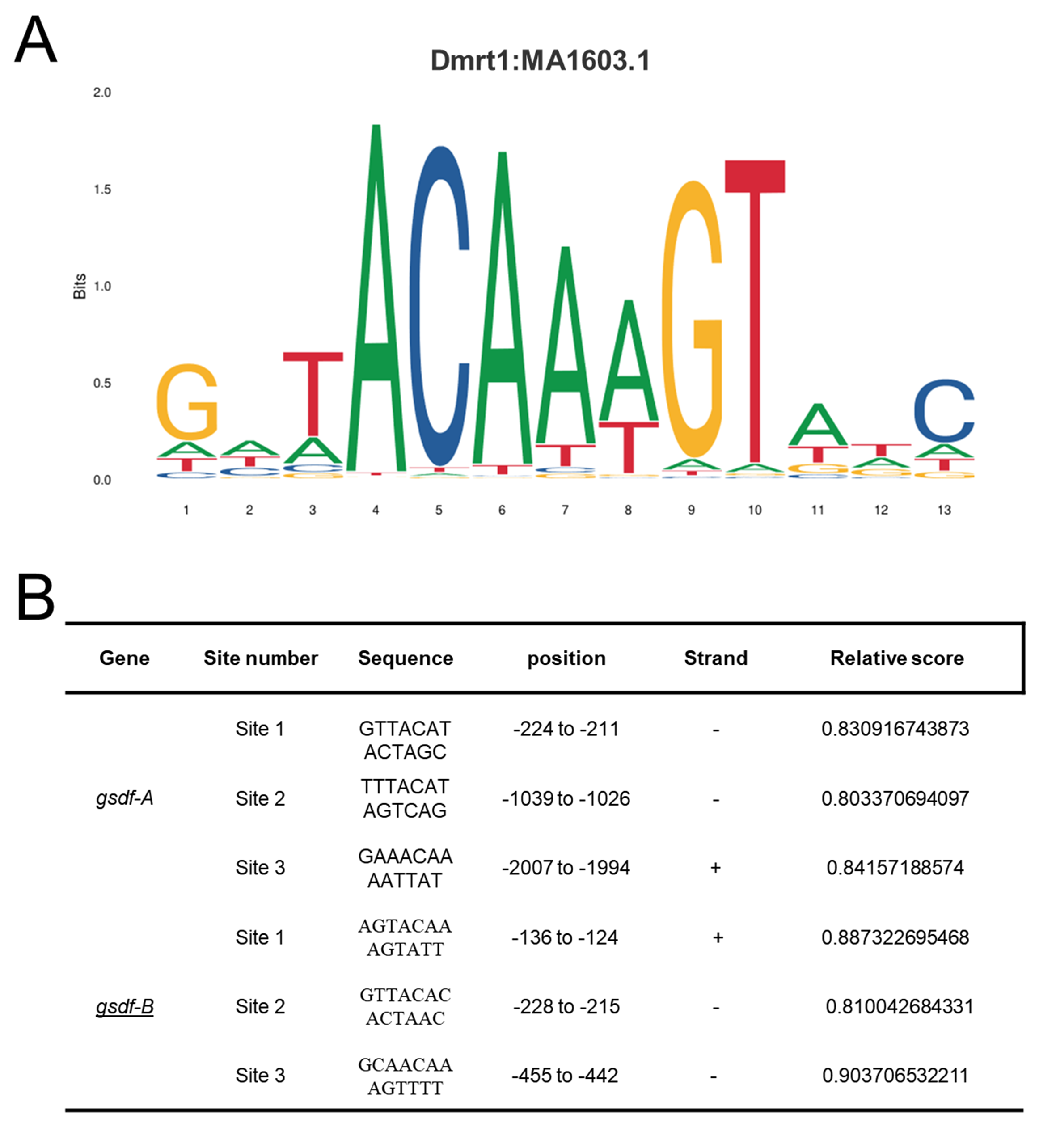

Supplement: S3 Fig — (A) Sequence preference of Dmrt1 recognition motif (MA1603.1) from JASPAR database. (B) Information about putative Dmrt1-binding sites of gsdf promoter. (TIF) [file pgen.1010288.s003.TIF]

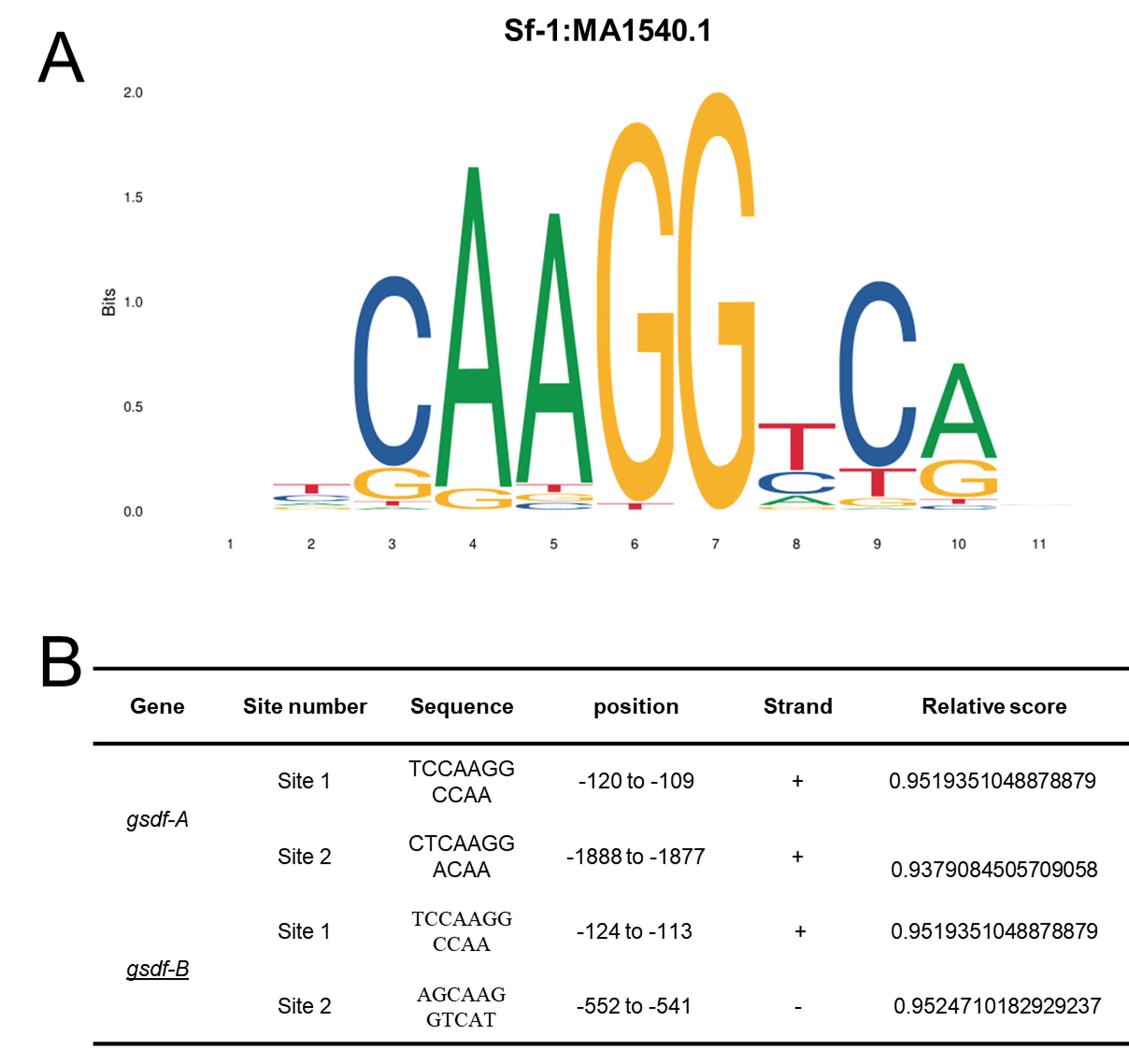

Supplement: S4 Fig — (A) Sequence preference of Sf1 recognition motif (MA1540.1) from JASPAR database. (B) Information about putative Sf1-binding sites of gsdf promoter. (TIF) [file pgen.1010288.s004.TIF]

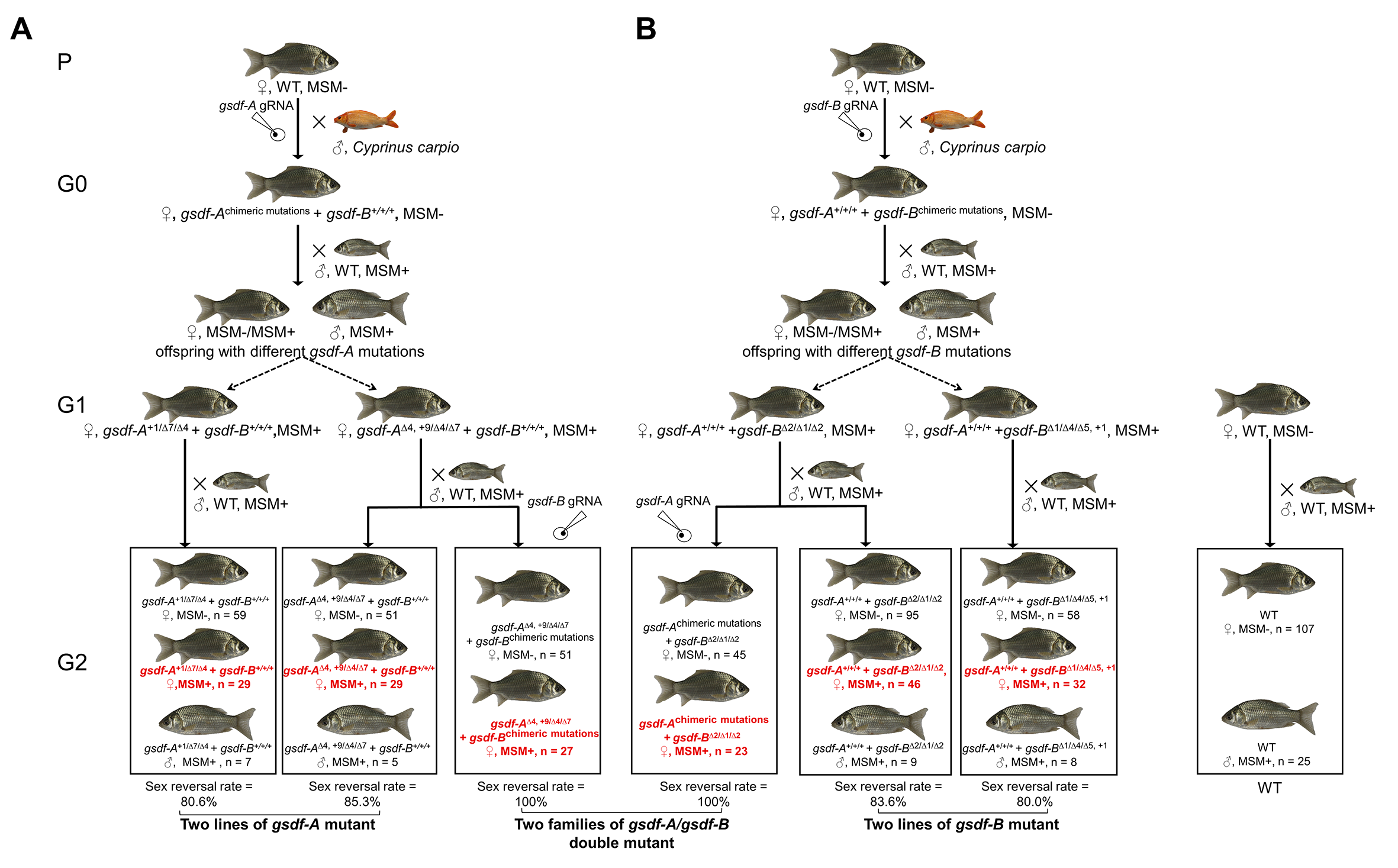

Supplement: S5 Fig — (A-B) Construction of different gsdf-A mutant lines (A), gsdf-B mutant lines (B), and gsdf-A/gsdf-B double mutant families (A, B). Sex reversed individuals are marked in red color and sex reversal rate was shown at the bottom of each line or family. ♀, phenotypic female; ♂, phenotypic male. (TIF) [file pgen.1010288.s005.tif]

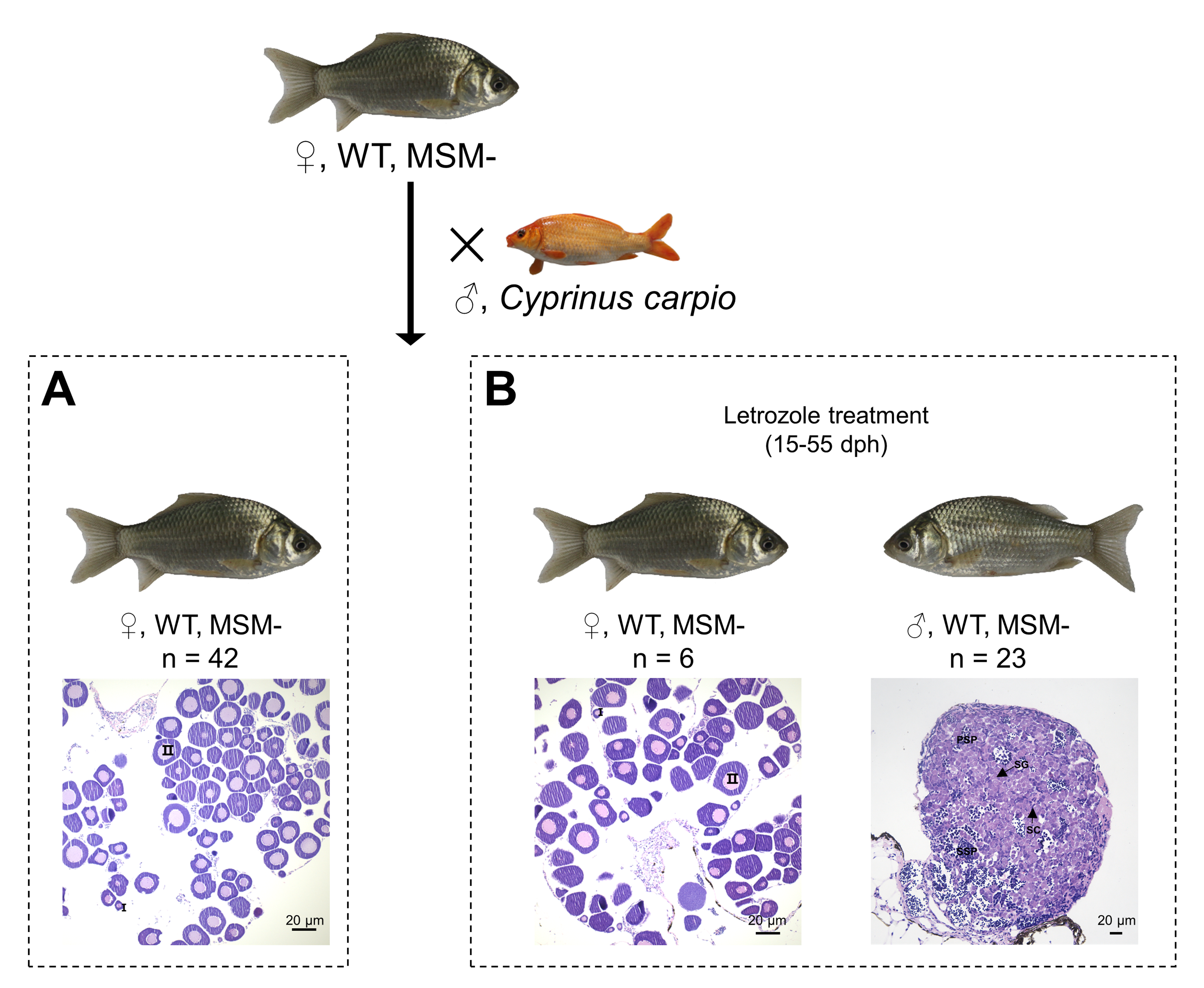

Supplement: S6 Fig — (A) Gonadal histology of gynogenetic offspring without letrozole treatment at 100 dph. (B) Gonadal histology of gynogenetic offspring with letrozole treatment at 100 dph. Bar: 20 μm. I, primary oocyte; II, growth stage oocyte; SG: spermatogonium; PSP: primary spermatocyte; SSP: secondary spermatocyte; SC: somatic cell. MSM−, without MSM; WT, wild type. (TIF) [file pgen.1010288.s006.tif]

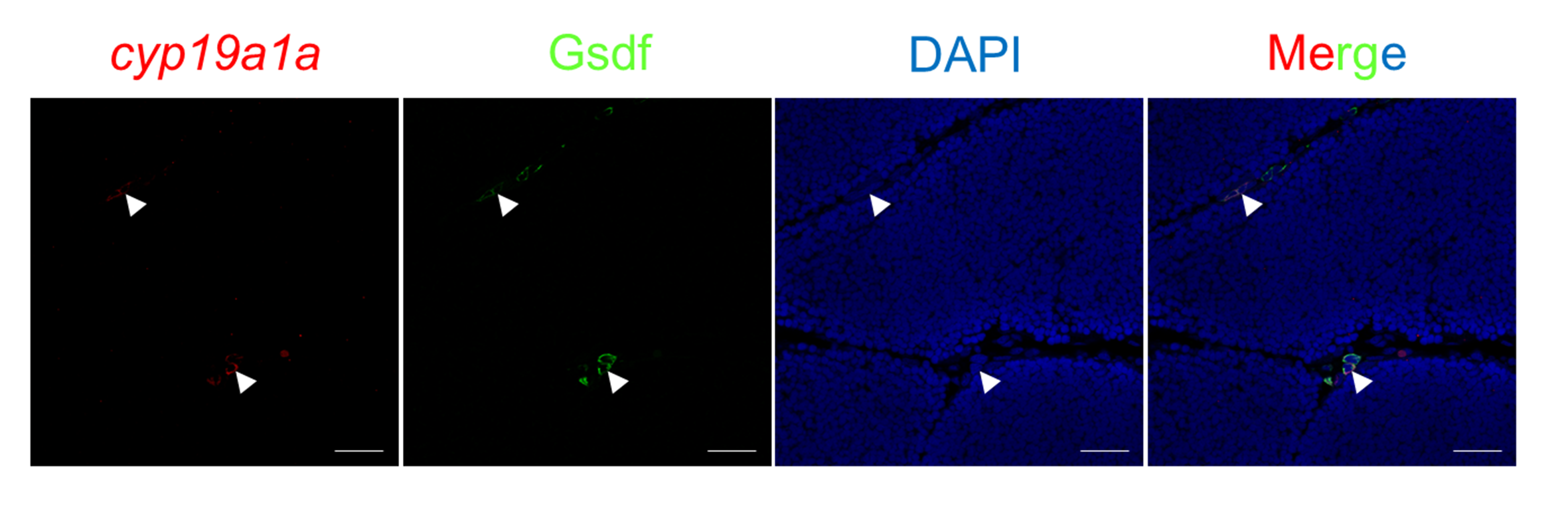

Supplement: S7 Fig — Arrowhead indicates the somatic cells with expression of cyp19a1a and Gsdf. Scale bars: 25 μm. (TIF) [file pgen.1010288.s007.TIF]

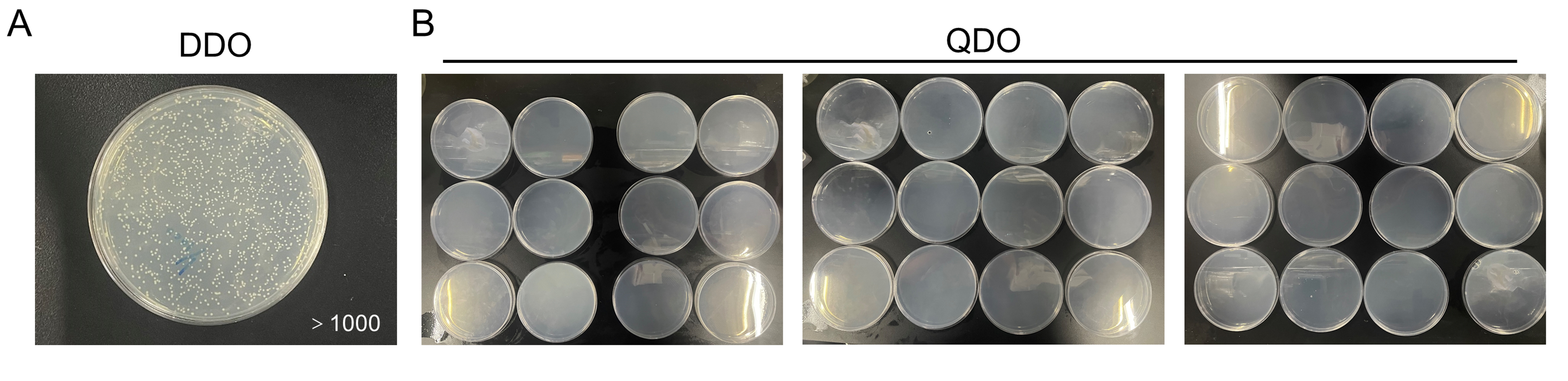

Supplement: S8 Fig — (A) Plate counting of 1,000-fold diluted yeast cells co-transformed with pBT3-SUC-Gsdf-A mature peptide and pPR3-N-library on DDO plate. The number of clones is displayed at the right bottom. (B) Undiluted yeast cells co-transformed with pBT3-SUC-Gsdf-A mature peptide and pPR3-N-library on QDO plate. No positive cells. (TIF) [file pgen.1010288.s008.tif]

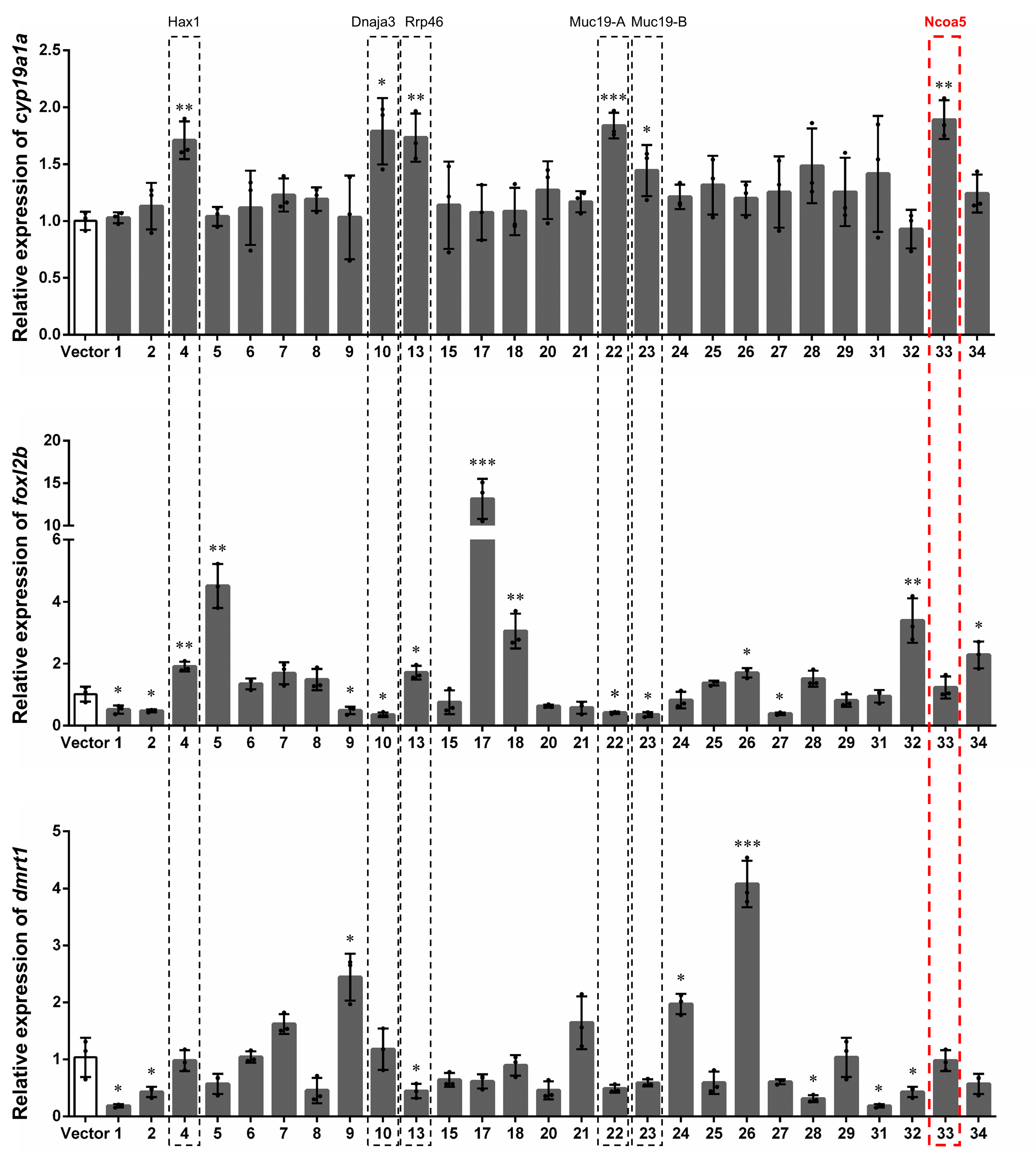

Supplement: S9 Fig — qPCR analysis of cyp19a1a, foxl2b, and dmrt1 expression in the CAB cells transfected with different plasmids. Candidate genes represented by different numbers are given in Fig 6B. Different letters represent statistical differences (*P<0.05, **P<0.01, ***P<0.001). Ncoa5 activated cyp19a1a transcription but could not change expression levels of foxl2b and dmrt1. (TIF) [file pgen.1010288.s009.tif]

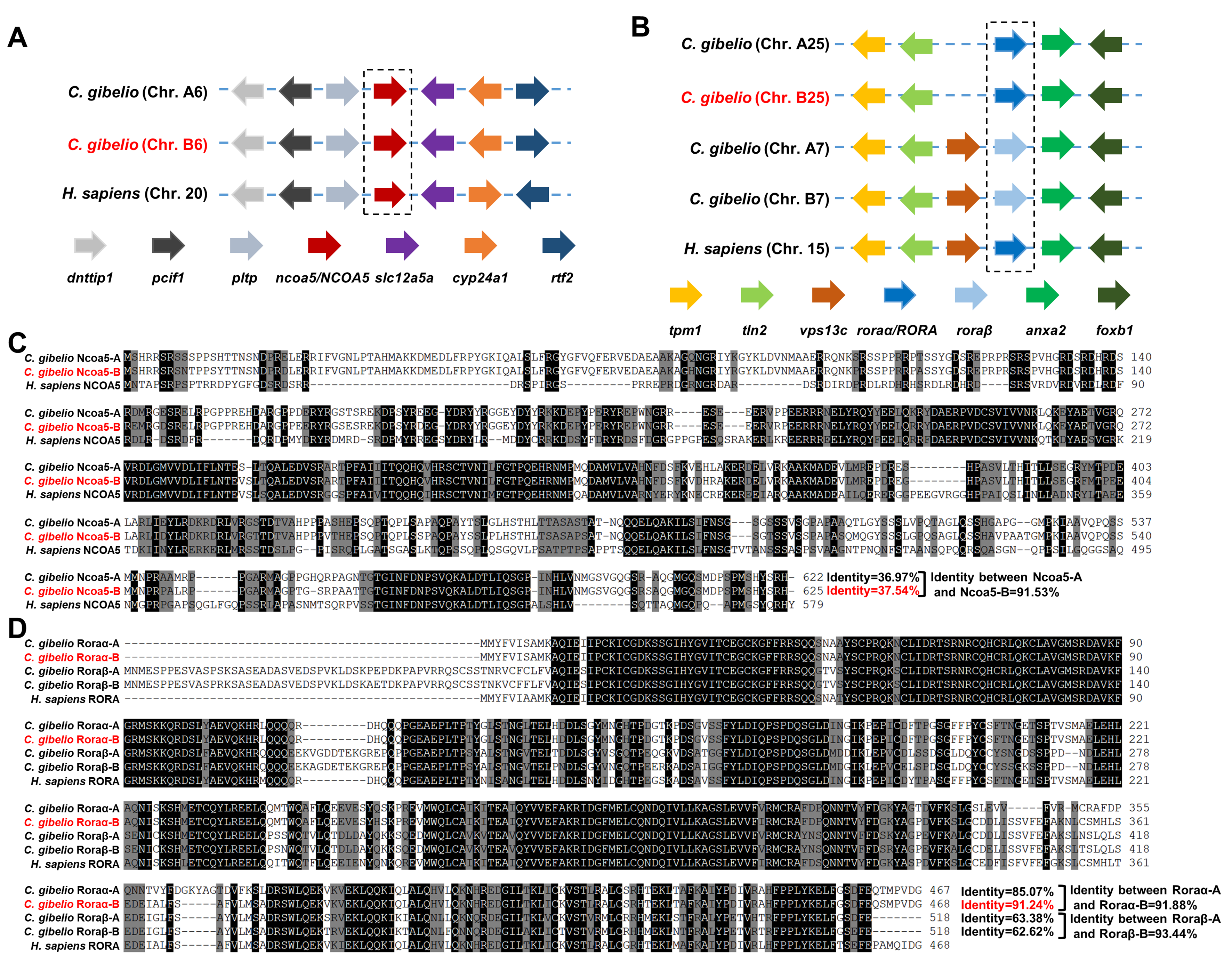

Supplement: S10 Fig — (A-B) Chromosomal localization of ncoa5 (A) and rora (B). Chromosome numbers are displayed at the left side. Conserved gene blocks are represented in matching colors. Transcription orientations are indicated by arrows. (C-D) Deduced amino acid sequence alignment of Ncoa5 (C) and Rora (D). The identities relative to human orthologs are exhibited at the end of each sequence. Ncoa5-B and Roraα-B were selected for subsequent in vitro analyses. (TIF) [file pgen.1010288.s010.tif]

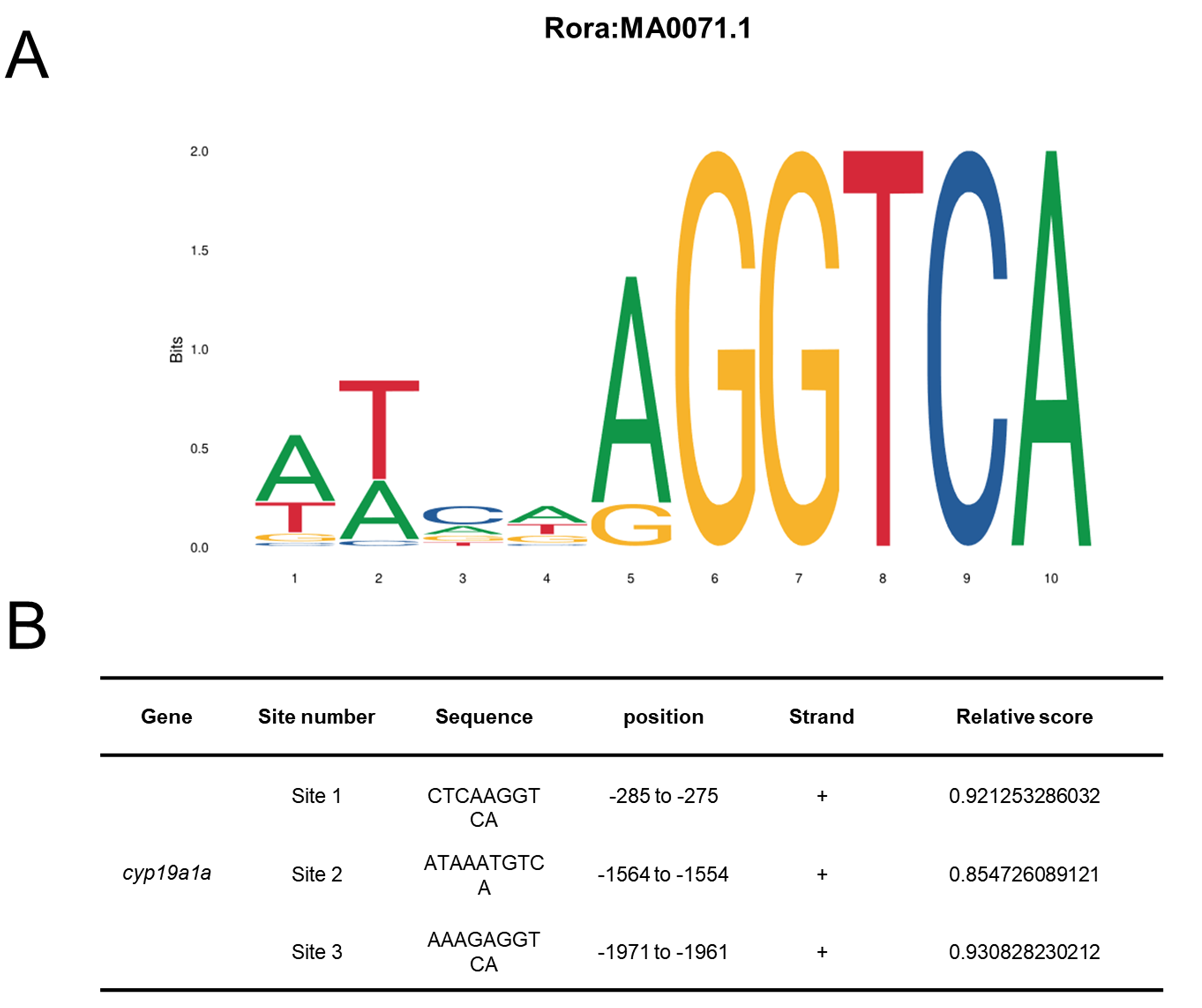

Supplement: S11 Fig — (A) Sequence preference of Rora recognition motif (MA0071.1) from JASPAR database. (B) Information about putative Rora-binding sites of cyp19a1a promoter. (TIF) [file pgen.1010288.s011.TIF]

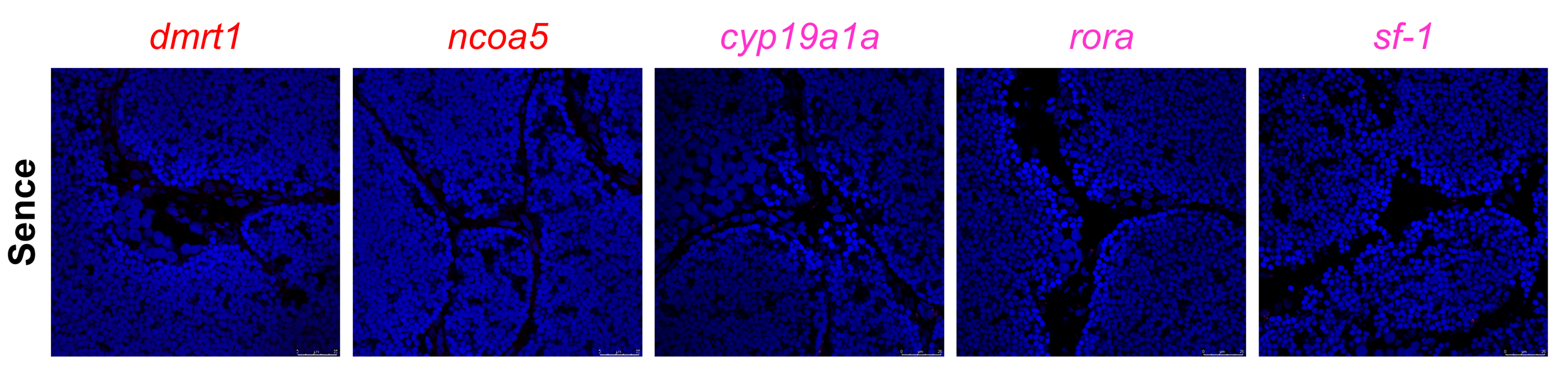

Supplement: S12 Fig — Sections of mature testis were subjected for FISH with sense riboprobes of dmrt1 (Red), ncoa5 (Red), cyp19a1a (Pink), roar (Pink), and sf-1 (Pink), and analyzed by fluorescence microscopy. Scale bars: 25 μm. (TIF) [file pgen.1010288.s012.tif]

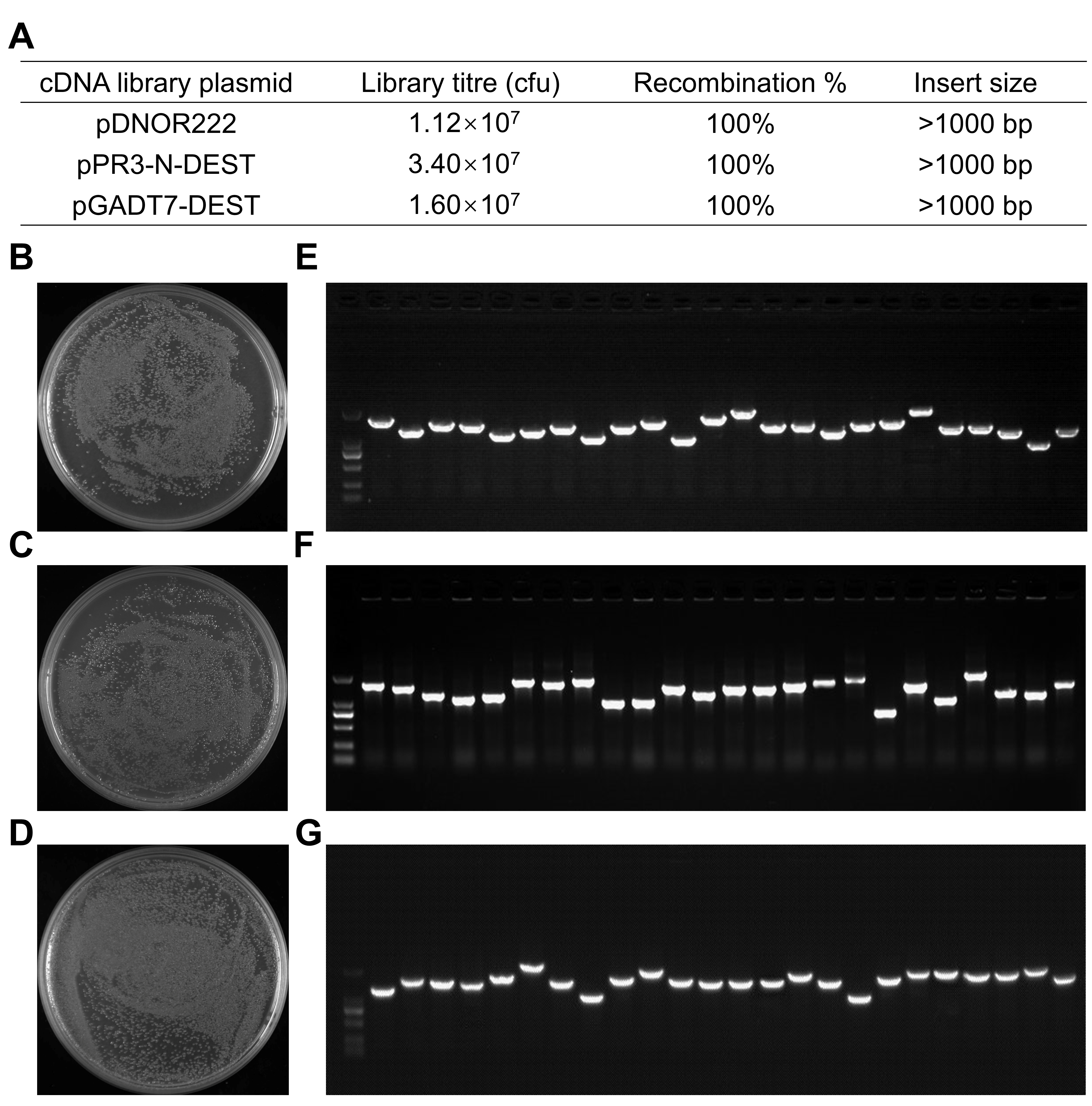

Supplement: S13 Fig — (A) Summary of three cDNA libraries using different plasmids including pDNOR222, pPR3-N-DEST, and pGADT7-DEST. (B-D) Plate counting of 200-fold diluted E. coli cells from the libraries of pDNOR222 (B), pPR3-N-DEST (C), and pGADT7-DEST (D). (E-G) Agarose gel electrophoresis of PCR products from randomly selected 24 colonies from the library of pDNOR222 (E), pPR3-N-DEST (F), and pGADT7-DEST (G). Marker is DL2000 DNA marker. (TIF) [file pgen.1010288.s013.tif]
