# Supplementary material for: Two duplicated gsdf homeologs cooperatively regulate male differentiation by inhibiting cyp19a1a transcription in a hexaploid fish
Source: PLoS Genet. 2022 Jun 29;18(6):e1010288. doi: 10.1371/journal.pgen.1010288 (PMC9275722; doi:10.1371/journal.pgen.1010288)
Supplement: S1 Table — (DOCX) [file pgen.1010288.s014.docx]

**S1 Table. Comparison of coding sequences and protein sequences between *gsdf-A* and *gsdf-B* alleles.**

| **Pairwise comparison** | | **Coding sequence identity** | **Protein sequence identity** | **Differing nucleotides** | **Differing nucleotides that cause amino acid changes (Ratio)** |
| --- | --- | --- | --- | --- | --- |
| *gsdf-A* allele 1 | *gsdf-B* allele 1 | 85.71% | 77.66% | 81 | 57 (57/81=70.37%) |
|  | *gsdf-B* allele 2 | 85.01% | 77.13% | 85 | 58 (58/85=68.24%) |
|  | *gsdf-B* allele 3 | 85.36% | 77.13% | 83 | 58 (58/83=69.88%) |
| *gsdf-A* allele 2 | *gsdf-B* allele 1 | 85.89% | 78.19% | 80 | 56 (56/80=70.00%) |
|  | *gsdf-B* allele 2 | 85.19% | 77.66% | 84 | 57 (57/84=67.86%) |
|  | *gsdf-B* allele 3 | 85.54% | 77.66% | 82 | 57 (57/82=69.51%) |
| *gsdf-A* allele 3 | *gsdf-B* allele 1 | 85.54% | 78.19% | 82 | 56 (56/82=68.29%) |
|  | *gsdf-B* allele 2 | 84.83% | 77.66% | 86 | 57 (57/86=66.28%) |
|  | *gsdf-B* allele 3 | 85.19% | 77.66% | 84 | 57 (57/84=67.86%) |
