# Supplementary material for: Two duplicated gsdf homeologs cooperatively regulate male differentiation by inhibiting cyp19a1a transcription in a hexaploid fish
Source: PLoS Genet. 2022 Jun 29;18(6):e1010288. doi: 10.1371/journal.pgen.1010288 (PMC9275722; doi:10.1371/journal.pgen.1010288)
Supplement: S2 Table — (DOCX) [file pgen.1010288.s015.docx]

**S2 Table. Primers used in this study.**

| **Primer name** | **Sequences (5′-3′)** |
| --- | --- |
| Detect-F-A | GCCAGTGTAGATAAATCCCTGC |
| Detect-R-A | GTCAGACGAGCACGATGAGGTCA |
| Detect-F-B | CTGGATATCTTCATAAAGCTTGTC |
| Detect-R-B | CATCTTCGACATGGACACACGAGG |
| *Gsdf*-5′ race-R1 | CATCTTCGACATGGACACACGAGG |
| *Gsdf*-5′ race-R2 | GGCTTTCACAGCAGATGCCTCAG |
| *Gsdf*-3′ race-F1 | CGTGAGTGCGGCTGTGACCC |
| *Gsdf*-3′ race-F2 | CAGCGGCCAGCGAATGTTCCC |
| *Gsdf-A*-ORF-F | ATGCCTCTGGTGTTGTGTGTCGTAG |
| *Gsdf-A*-ORF-R | CTAAGACAGGGCCATGACCTCAG |
| *Gsdf-B*-ORF-F | ATGCCTCTGGTGCTGTGTGTCGTC |
| *Gsdf-B*-ORF-R | CTAGGACGGGGTCATGACCTCTG |
| *Gsdf-A*-gRNA-F | GTAATACGACTCACTATAGACAGCAGATGCCTCAGGTGTGTTTTAGAGCTAGAAATAGC |
| *Gsdf-B*-gRNA-F | GTAATACGACTCACTATAGGCAAGGGAGACCTGGAAATCGTTTTAGAGCTAGAAATAGC |
| gRNA-R | AAAAGCACCGACTCGGTGCC |
| RT-*dmrt1-A*-F | CATCATCATCATCACCTGTCGG |
| RT- *dmrt1-A*-R | TTAGAGCCAGCATGGAGCGATT |
| RT- *dmrt1-B*-F | GCTGCAGCATCACCGTTATCATC |
| RT- *dmrt1-B*-R | CTGTCTTTCAGCGATCAGTCTGC |
| RT-*gsdf-A*-F | CCTACACCTGAGGCATCTGCTGTG |
| RT-*gsdf-A*-R | GTTACCTCCTGGTGAGGATGGCGT |
| RT-*gsdf-B*-F | CCTTCACCTGAGGCATCTGCTGTC |
| RT-*gsdf-B*-R | GTTACCTGCTGGTGCTGATGTGAG |
| RT-*gsdf*-F | CCTCGTGTGTCCATGTCGAAGATG |
| RT-*gsdf*-R | GTGATGCTCTCTGGATAAACCACCC |
| RT-*dmrt1*-F | GAAATGGTCGCTATAACGTG |
| RT-*dmrt1*-R | GATGATGGAGTCGACAGAGA |
| RT-a*mh*-F | GATTTTGGCTGTTGCTGATTGT |
| RT-*amh*-R | CCTTGGGAACGTGAGGGTTA |
| *RT-foxlb2*-F | CGCGGTGAAGAAAGAAGAGTTCGC |
| *RT-foxl2b*-R | AGGTTGTGTCGGATGCTGTTCTGC |
| RT-*cyp19a1a*-F | CCATCCGGTCGTGGACTTCAC |
| RT-*cyp19a1a*-R | TGTGCATCCGACCCACGTTCAG |
| RT-*piwil*-F | GATTGGACGCCATTACTACAAC |
| RT-*piwil*-R | TCTGCCCTCTCTCTGTTCAG |
| RT-*dnd*-F | GAACTTCAGTGGGCAGAACC |
| RT-*dnd*-R | GTCAGAGATCATTCGCAGCA |
| RT-*ncoa5*-F | TGGTGGTGAATCTCATCTTCC |
| RT-*ncoa5*-R | CCTTTCTTTAGCACGGTGTTC |
| RT-*rora*-F | GCCCAGAACATTTCCAAGTC |
| RT-*rora*-R | CTGATAAACACCACCTCCAAAG |
| RT-*β-Actin*-F | AGCACGGTATTGTGACTAACTG |
| RT-*β-Actin*-R | TCGAACATGATCTGTGTCATC |
| pGADT7-F (T7) | TAATACGACTCACTATAGGGCGAGCGCCGCCATG |
| pGADT7-R (ADR) | GTGAACTTGCGGGGTTTTTCAGTATCTACGATT |
| Chip-*rora*-F | GGGATGTGGAATGACTCAAG |
| Chip-*rora*-R | TCAAGACACGCAAACCTTC |
| *sf1*-sense-F | TAATACGACTCACTATAGGGCATAACTGCTGGAGTGAGC |
| *sf1*-sense-R | GAGGATGAGGAACTTCAGACAG |
| *sf1*-antisense-F | GCATAACTGCTGGAGTGAGC |
| *sf1*-antisense-R | TAATACGACTCACTATAGGGAGGATGAGGAACTTCAGACAG |
| *cyp19a1a* -sense-F | TAATACGACTCACTATAGGCATCTTGAGCAGGTCGTCTG |
| *cyp19a1a* -sense-R | TCCAAAGTTCTCTGAAGCCC |
| *cyp19a1a* -antisense-F | CATCTTGAGCAGGTCGTCTG |
| *cyp19a1a* -antisense-R | TAATACGACTCACTATAGGTCCAAAGTTCTCTGAAGCCC |
| *dmrt-1*-sense-F | TAATACGACTCACTATAGGTCTACCAGCCCACACCATAC |
| *dmrt-1*-sense-R | ACACTCCAGCCTGTTCTCAG |
| *dmrt-1*-antisense-F | TCTACCAGCCCACACCATAC |
| *dmrt-1*-antisense-R | TAATACGACTCACTATAGGACACTCCAGCCTGTTCTCAG |
| *roraα*-sense-F | TAATACGACTCACTATAGGACTGTTCCTTCACCAATGGG |
| *roraα*-sense-R | TGGCAAACTCCACCACATAC |
| *roraα*-antisense-F | ACTGTTCCTTCACCAATGGG |
| *roraα*-antisense-R | TAATACGACTCACTATAGGTGGCAAACTCCACCACATAC |
| *ncoa5*-sense-F | TAATACGACTCACTATAGGTCATCCTCAGTGTCTGGTCC |
| *ncoa5*-sense-R | AGCGTATCCAAGGCTTTCTG |
| *ncoa5*-antisense-F | TCATCCTCAGTGTCTGGTCC |
| *ncoa5*-antisense-R | TAATACGACTCACTATAGGAGCGTATCCAAGGCTTTCTG |
