# Supplementary material for: Two duplicated gsdf homeologs cooperatively regulate male differentiation by inhibiting cyp19a1a transcription in a hexaploid fish
Source: PLoS Genet. 2022 Jun 29;18(6):e1010288. doi: 10.1371/journal.pgen.1010288 (PMC9275722; doi:10.1371/journal.pgen.1010288)
Supplement: Attachment — Submitted filename: Response to Reviewers.docx [file pgen.1010288.s016.docx]

Dear Dr. Schartl and Dr. Barsh,
Thank you very much for your efforts in handling our manuscript “Two duplicated *gsdf* homeologs cooperatively regulate male differentiation by inhibiting *cyp19a1a* transcription in a hexaploid fish” (PGENETICS-D-22-00317). Based on the helpful comments and suggestions of three reviewers, we substantially revised our manuscript by adding new experimental data, explaining the subgenome-specific regulation of *gsdf*, and providing the screening process of Gsdf’s potential interaction partners. All the revised parts are indicated in red in the texts to make them easy to check. Point-by-point responses to the reviewers’ comments are provided below.

**Reviewer #1:**

Two duplicated gsdf homeologs cooperatively regulate male differentiation by inhibiting cyp19a1a transcription in a hexaploid fish.
In their study, Wang et al. have identified two gsdf homeologous genes in the hexaploid gibel carp. They further propose to better characterize their regulation and function regarding to gonad development.
 First, having in mind to decipher how duplicated genes co-regulate a biological process (gonadal development for instance) in a polyploid species, they show that the transcription of gsdfA and gsdfB is mainly activated by dmrt1a and dmrt1b respectively, suggesting asymmetrical evolution to explain the expression divergence in the context of the two sub-genomes. Then, functional characterization is carried on through generation of gsdf mutants, leading to partial or total sex reversion. The cooperative ability of the two gsdf homeologs to regulate male differentiation is then shown. Using cell culture system, Gsdf interactions with Ncoa5 and subsequent transcriptional modulation of Cyp19a1a is further demonstrated.
 All in one, although the whole story presented in this paper draft is interesting in terms of (i) evolutionary biology (how duplicated genes co-regulate a biological process, and how asymmetrical evolution rule expression divergence), and (ii) mechanistic/physiology (how gsdfs exert their function(s)), I think some major issues need to be addressed before this draft is ready for publication in PLoS Genetics.
**Response:** Thank you very much for your positive comments on this work.

Major Issues:
**1.** Characterization of gsdf homeologs and alleles/dynamic transcription of gsdfA/B are mainly activated by dmrt1A/B respectively.
Co-evolution of the two subgenomes (regarding to gsdfs regulation by Dmrt1) is indeed an interesting aspect of that paper.
-The analysis might benefit from a dN/dS analysis (gsdf and dmrt1) to check whether one or the other homeologs are under selection. This might tell us more about the co-evolution. It should be then discussed if necessary.

**Response:** Thanks for your comments and suggestions. We have added co-localization and dN/dS analyses of *gsdf* and *dmrt1* genes in the revised manuscript: “*Fluorescence in situ hybridization (FISH) analysis showed that dmrt1 and sf1 mRNA were co-expressed in some Gsdf positive somatic cells in testis (Fig 3G). In addition, we performed dN/dS analyses of dmrt1 and gsdf genes to check whether one or the other homeologs are under selection. Using D. rerio as reference, the dN/dS value of dmrt1-A was significant lower than that of dmrt1-B (χ^2^ test p value: 7.93×10^−4^) under the two-ratio model (Fig 3H and 3J). On the other side, the dN/dS analysis of gsdf homeolgs met one-ratio model and display the same value (Fig 3I and3 K). These results indicate that the dmrt1 and gsdf homeologs are under asymmetric and symmetric purifying selection, respectively. Thus, the differential expression between gsdf-A and gsdf-B might be resulted from the divergent evolution of dmrt1-A and dmrt1-B.*” (line 233-243 in the revised manuscript)

**Revised Fig 3. Dmrt1 regulates *gsdf* transcription in the presence of Sf-1.**

**(G)** FISH analysis of the *dmrt1*(Red) and *sf-1* (Pink) mRNA and, immunofluorescence analysis of the Gsdf protein (Green) in mature testis. Arrowhead indicates the somatic cells with expression of *sf-1*, *dmrt1*, and Gsdf. Scale bars: 25 µm. **(H-I)** Phylogenetic analysis of *dmrt1* **(H)** and *gsdf* **(I)** coding sequences from different fish species. Phylogenetic analysis was performed using the Neighbor-Joining method. The percentage of replicate trees in which the associated taxa clustered together in the bootstrap test (2000 replicates) are shown next to the branches. The tree is drawn to scale, with branch lengths in the same units as those of the evolutionary distances used to infer the phylogenetic tree. The evolutionary distances were computed using the Nei-Gojobori method and are in the units of the number of synonymous differences per sequence. The analysis involved 7 nucleotide sequences. All positions containing gaps and missing data were eliminated. **(J-K)** Information of dN/dS analysis of *dmrt1A/B* **(J)** or *gsdfA/B* **(K)**. The mode of each gene pairs is selected according to the *P* value (χ^2^ test), which is marked in bold. The *dmrt1* or *gsdf* of *D. rerio* were served as references.

-In silico analysis: authors need to tell us more about the predicted dmrt1/sf1-binding sites (which matrix was employed, sequence, homology, divergence to the canonical sequence…)

**Response:** Thanks for your suggestions and we have added the information of Dmrt1/Sf1-binding sites in the revised Figure S3 and S4 (line 207-208 in the revised manuscript).

**Revised S3 Fig. Sequence preference of Dmrt1 recognition motif and putative Dmrt1-binding sites.**

**(A)** Sequence preference of Dmrt1 recognition motif (MA1603.1) from JASPAR database. **(B)** Information about putative Dmrt1-binding sites of *gsdf* promoter.

**Revised S4 Fig. Sequence preference of Sf1 recognition motif and putative Sf1-binding sites.**

**(A)** Sequence preference of Dmrt1 recognition motif (MA1540.1) from JASPAR database. **(B)** Information about putative Sf1-binding sites of *gsdf* promoter.

-At the end, we need to know if this differential regulation is due to the evolution (divergence) of the Dmrt1s only, or of the targets only, or co-evolution of both.
**Response:** According to results of the dN/dS analyses in the revised Figure 3 (Fig 3H-3K, showed above), we find out that the *dmrt1* and *gsdf* homeologs are under asymmetric and symmetric purifying selection, respectively. Thus, the differential expression between *gsdf-A* and *gsdf-B* might be resulted from the divergent evolution of *dmrt1-A* and *dmrt1-B* (line 235-243 in the revised manuscript).

-Cytoplasmic localization of the Gsdf proteins is OK for me, although we know it is a secreted protein that will diffuse from one cell to others, and bind at cell membranes. It might accumulate in some cells before being excreted; this should be told and discussed.

**Response:** Thanks for your advice. We have added this information in the text as follows: “*Along with spermatogenesis, the green signal derived from Gsdf was mostly distributed in the cytoplasm of somatic cells surrounding the germ cells (Fig 2C), which was consistent with the main distribution in* *Sertoli cells of other fishes (**Chen et al., 2016**;* *Kaneko et al., 2015**;* *Myosho et al., 2012**;* *Shibata et al., 2010**). Gsdf, as a member of the TGF-β superfamily, is usually considered to be a secreted ligand that will band to its cell surface receptors (**Derynck and Budi, 2019**;* *Zhang et al., 2017**). However, Gsdf proteins might also accumulate in cytoplasm before being excreted.*” (line 201-206 in the revised manuscript)

-Disruption of gsdfA/B results in cyp19a1a upregulation and aromatase inhibitor treatment rescues the male to female sex reversal.
That part is OK for me.

**Response:** Thank you very much for your positive comments.

-Figure 3A/3B is very complex and I am not sure it helps much here. I would move it to supplemental data.

**Response:** Thanks for your advice and we have moved the original Figure 3A/3B to supplemental data as revised Figure S5.

-Figure 4E: it is difficult to compare that figure 4E with what has been shown in figure 2B: in 2B, basal relative expression of Gsdfs is nearly not detectable (at 25 dph), while that basal expression is quite high in 4E (same stage). Reference to (E) is actually not well written in the figure legend.
So, please unify the relative expressions (2B/4E) or explain that discrepancy.
**Response:** We apologize for the confusing exhibitions in Figure 2B and 4E. For relative qPCR analysis, *β-actin* was used as internal reference and relative expression level of target gene was calculated with 2^-ΔΔCT^ method. In each analysis, the highest expression level was used as control and defined as 1. Thus, different relative qPCR analyses have different controls. In the original Figure 2B, the expression of *gsdf-A* at 250 dph was used as control and defined as 1. Since the expression level of *gsdf-A* at 250 dph was so high that the basal relative expressions of *gsdf-A* and *gsdf-B* in male gonads at 21 and 30 dph appeared to be low. In the original Figure 4E, the *gsdf-A* expression in *gsdf-B* mutants was used as control and defined as 1 in the analysis of *gsdf-B* mutants, while the *gsdf-B* expression in *gsdf-A* mutants was used as control and defined as 1 in the analysis of *gsdf-A* mutants. As the expression levels of these two controls were not high, the basal relative expressions of *gsdf-A* and *gsdf-B* in wild type male gonads at 25 dph appeared to be quite high.

Thus, we have revised the Materials and Methods section as follows “*The highest expression level in each qPCR analysis was used as control and defined as 1 separately.*” (line 563-565 in the revised manuscript). And we also added control definition of relative qPCR analyses in the corresponding figure legends (line 1073-1074, 1118-1119, 1129-1130, and 1174-1175).

In addition, we have also rewritten the figure legend of the original Figure 4E: “*Gsdf-A compensatory expression in the gonads of gsdf-B mutants and gsdf-B compensatory expression in the gonads of gsdf-A mutants at 25 dph.*” (line 1111-1113 in the revised manuscript).

The rest is OK for me, although at one point you will need RNA in situs to show that Gsdf, Ncoa5 and rora are indeed co-expressed to validate the physiological relevance of the inferred interactions.

**Response:** Thanks for your comments and suggestions. We have performed co-localization analysis of *rora*, *ncoa5*, and Gsdf and added this result into the revised Figure 8B: “*FISH analysis showed that rora and ncoa5 mRNA were co-expressed in some Gsdf positive somatic cells of mature testis (Fig 8B).*” (line 425-426 in the revised manuscript).

**Revised Fig 8B. FISH analysis of the *rora* (Pink) and *ncoa5* (Red) mRNA, and immunofluorescence analysis of the Gsdf protein (Green) in mature testis.** Arrowhead indicates the somatic cells with expression of *rora*, *ncoa5*, and Gsdf. Scale bars: 25 µm.

-Identification of GSDF-NCOa5 interaction via yeast two hybrid assay and co-immunoprecipitation.
-Well, although I could be at first convinced by the yeast two hybrid assay, I am nevertheless very concerned about the fact that the full open reading frame of Gsdf was used as a bait. Indeed, if I am right the whole ORF usually encodes for the pro-mature peptide. Then to be active the pro-domain has to be cleaved to release the mature gsdf active peptide. In these conditions, using the un-cleaved Gsdf, I am not certain that the found interactions are relevant. This is an issue.

-The same question now stands for the antibody that was prepared from (most of) the whole sequence; and that might also recognize the pro-domain instead of the mature gsdf peptide.

**Response:** Thanks for your comments. As a member of TGF-β superfamily, Gsdf commonly is considered to be a ligand to bind to its cell surface receptors. The precursor molecules of TGF-β family proteins usually consist of a signal peptide, a prodomain, and the mature polypeptide. After removal of signal peptide, the prodomain is cleaved to release the mature active peptide. We tried to identify potential interaction membrane proteins of secreted Gsdf mature peptide via DUAL membrane system but failed. Then, we have identified several potential interaction partners of Gsdf-A precursor molecule via GAL4 system.

We have added the screening process of DUAL membrane system in the revised manuscript as follows: “*To identify potential interaction membrane proteins of secreted Gsdf, the coding sequence of Gsdf-A mature peptide was cloned into* *pBT3-SUC (pBT3-SUC-Gsdf-A* *mature peptide) as a bait to perform yeast two hybrid assay via DUAL membrane system (Dualsystems Biotech). However, screens did not yield any interactors (S8 Fig).*” (line 354-358 in the revised manuscript).

**Revised S8 Fig. Yeast two-hybrid assay using DUAL membrane system**

**(A)** Plate counting of 1,000-fold diluted yeast cells co-transformed with pBT3-SUC-Gsdf-A mature peptide and pPR3-N-library on DDO plate. The number of clones is displayed at the right bottom. **(B)** Undiluted yeast cells co-transformed with pBT3-SUC-Gsdf-A mature peptide and pPR3-N-library on QDO plate. No positive cell grows on QDO plate.

Besides, we analyzed the interaction between Gsdf-A mature peptide and TGF-β type II receptors including Tgfbr2a-A, Tgfbr2b-A, and Bmpr2a-A. The yeast co-transformed with pBT3-SUC-Gsdf-A mature peptide + pPR3-N-Tgfbr2a-A, pBT3-SUC-Gsdf-A mature peptide + pPR3-N-Tgfbr2b-A, and pBT3-SUC-Gsdf-A mature peptide + pPR3-N-Bmpr2a-A grow on TDO (lack of His, Leu, and Trp) plates but did not grow on QDO (lack of Ade, His, Leu, and Trp) plates, indicating that there were only very weak interaction between Gsdf-A mature polypeptide and these receptors (Figure R1).

**Figure R1 Yeast-two-hybrid assays for bait (Gsdf-A mature peptide) interaction with the prey (Tgfbr2a-A , Tgfbr2b-A and Bmpr2a-A).** 
Yeast cells co-transformed with bait and prey plasmids that survived on TDO and QDO plate indicate weak and strong positive interactions, respectively. The pBT3-SUC-Gsdf-A + pPR3-N was used for detection of self-activation, while the pBT3-SUC-Gsdf-A + POST-Nubal was used for functional verification of the ubiquitin system. The pTSU2-APP + pPR3-N and pTSU2-APP + pNubG-Fe65 were used as the negative and positive control, respectively.

Gsdf-Ncoa5 interaction has been demonstrated by yeast two hybrid assay and co-immunoprecipitation (original Figure 5). The interaction between Gsdf and Ncoa5 could regulate *cyp19a1a* transcription in tissue culture cells (original Figure 7). Besides, we have added the co-localization analysis and revealed that *rora* and *ncoa5* mRNA are co-expressed in some Gsdf positive somatic cells of mature testis. (revised Figure 8B). Thus, we believe that Gsdf might have functions in cells, which is independent of ligand-receptor pathway.

The antibody of Gsdf was prepared from most of the amino acid sequence and that might recognize the pro-domain instead of the mature Gsdf peptide. Based on the response above, we believe the unsecreted Gsdf protein might exercise function in a non-ligand/receptor manner.

**Revised Fig 8B. FISH analysis of the *rora* (Pink) and *ncoa5* (Red) mRNA, and immunofluorescence analysis of the Gsdf protein (Green) in mature testis.** Arrowhead indicates the somatic cells with expression of *rora*, *ncoa5*, and Gsdf. Scale bars: 25 µm.

-NCOa5 participates in cyp19a1a regulation via interaction with Rora.
-Like for Sf1 and Dmrt1 predicted binding sites, potential Rora binding sites should be described with more details: statistics concerning the homology to the matrix and so on.
The rest of that part seems OK for me.
**Response:** Thanks for your suggestions. We have added the information of Rora-binding sites in the revised Figure S11。

**Revised S11 Fig. Sequence preference of Rora recognition motif and putative Rora-binding sites.**

**(A)** Sequence preference of Rora recognition motif (MA0071.1) from JASPAR database. **(B)** Information about putative Rora-binding sites of *cyp19a1a* promoter.

-Ncoa5 participates in cyp19a1a regulation via interaction with Rora.
-Regulations and interactions were established in cells lines after transfection experiments. While the experiments performed for that part are quite OK, we should not forget the physiology behind, and how to interpret these results “physiologically”.

**Response:** Thanks for your comments and suggestion. To interpret these results physiologically, we have added FISH analysis and revealed that *rora* and *ncoa5* mRNA were co-expressed in Cyp19a1a positive somatic cells of mature testis (Fig 7F), supporting the conclusion that Rora regulates *cyp19a1a* transcription in presence of Ncoa5.

**Revised Fig 7F. FISH analysis of the *rora* (Pink) and *ncoa5* (Red) mRNA, and immunofluorescence analysis of the Cyp19a1a protein (Green) in mature testis.** Arrowhead indicates the somatic cells with expression of *rora*, *ncoa5*, and Cyp19a1a. Scale bars: 25 µm.

Indeed, while Figure 8 compile the whole story, it is difficult for me to understand how a TGF-beta ligand, that is an excreted molecule that usually binds at the cell surface, would here have an activity inside of the nucleus together with Ncoa5 and Rora. To my knowledge this is impossible.

Then to make the necessary connexion between the results in cell lines together with what the physiology would allow, it is to my opinion necessary to show co-localization of the Gsdf-Ncoa5-Rora complexes inside of the nucleus.

**Response:** Thanks for your comments. We are sorry that we misled you into thinking that the Gsdf protein has an activity inside of the nucleus together with Ncoa5 and Rora. Based on the cytoplasm distribution of Gsdf proteins (Original Figure 2C) and the *in vitro* results (Original Figure 5 and 7), we supposed that the Gsdf protein (precursor) might interact with Ncoa5 in cytoplasm, leading to the reduction of Ncoa5 that can interact with Rora.

Intrigued by your suggestion, we have performed the co-localization analysis of *rora*, *ncoa5*, and Gsdf, and found out that *rora* and *ncoa5* mRNA are co-expressed in some Gsdf positive somatic cells of mature testis (Fig 8B). These results support the hypothesis that Gsdf might interact with Ncoa5 in cytoplasm to reduce Ncoa5 entering the nucleus.

**Revised Fig 8B. FISH analysis of the *rora* (Pink) and *ncoa5* (Red) mRNA, and immunofluorescence analysis of the Gsdf protein (Green) in mature testis.** Arrowhead indicates the somatic cells with expression of *rora*, *ncoa5*, and Gsdf. Scale bars: 25 µm.

**Reviewer #2:**

Gsdf is a member of transforming growth factor β (TGF-β) superfamily which has been implicated in male sex differentiation of multiple teleost species. Despite of its critical role in testis differentiation, the specific molecular pathway of Gsdf-mediated sex differentiation remains elusive. In this MS, the authors identified two duplicated gsdf homeologs genes (gsdf-A and gsdf-B) in the gynogenetic hexaploid gibel carp, and found that the transcription of gsdf-A and gsdf-B is activated by dmrt1-A and dmrt1-B, respectively. By loss-of-function experiments, the authors demonstrated that two gsdf homeologs cooperatively regulate male differentiation by inhibiting cyp19a1a transcription. By in vitro analyses, the authors demonstrated that Gsdf-A and Gsdf-B interact with Ncoa5 to block Ncoa5 interaction with Rora, inhibiting Rora/Ncoa5-induced activation of cyp19a1a.
Generally, this study is interesting and provides a special mechanism of gsdf in regulating male sex determination and differentiation. However, the following issues should be addressed before the article is accepted.

**Response:** Thank you very much for your positive comments.

Major concerns:
1) As we know, Gsdf is a secretory protein which belongs to the transforming growth factor β family, and it is supposed to function with its receptor. In this MS, the author demonstrated a direct interaction between Gsdf-A/B and Ncoa5. Is there a TGF- β type II receptor involved in this regulatory process by binding gsdf?

**Response:** Thanks for your comments. As a member of TGF-β superfamily, Gsdf commonly is considered to be a ligand to bind to its cell surface receptors. We first tried to identify potential interaction membrane proteins of secreted Gsdf mature peptide via DUAL membrane system but failed. Then, we have identified several potential interaction partners of Gsdf-A precursor molecule via GAL4 system.

We have added the screening process of DUAL membrane system in the revised manuscript as follows: “*To identify potential interaction membrane proteins of secreted Gsdf, the coding sequence of Gsdf-A mature peptide was cloned into pBT3-SUC (pBT3-SUC-Gsdf-A mature peptide) as a bait to perform yeast two hybrid assay via via DUAL membrane system (Dualsystems Biotech). However, screens did not yield any interactors (S8 Fig).*” (line 354-358 in the revised manuscript).

**Revised S8 Fig. Yeast two-hybrid assay using DUAL membrane system**

**(A)** Plate counting of 1,000-fold diluted yeast cells co-transformed with pBT3-SUC-Gsdf-A mature peptide and pPR3-N-library on DDO plate. The number of clones is displayed at the right bottom. **(B)** Undiluted yeast cells co-transformed with pBT3-SUC-Gsdf-A mature peptide and pPR3-N-library on QDO plate. No positive cell grows on QDO plate.

Besides, we analyzed the interaction between Gsdf-A mature peptide and TGF-β type II receptors including Tgfbr2a-A, Tgfbr2b-A, and Bmpr2a-A. The yeast co-transformed with pBT3-SUC-Gsdf-A mature peptide + pPR3-N-Tgfbr2a-A, pBT3-SUC-Gsdf-A mature peptide + pPR3-N-Tgfbr2b-A, and pBT3-SUC-Gsdf-A mature peptide + pPR3-N-Bmpr2a-A grow on TDO (lack of His, Leu, and Trp) plates but did not grow on QDO (lack of Ade, His, Leu, and Trp) plates, indicating that there were only very weak interaction between Gsdf-A mature polypeptide and these receptors (Figure R1).

**Figure R1 Yeast-two-hybrid assays for bait (Gsdf-A mature peptide) interaction with the prey (Tgfbr2a-A , Tgfbr2b-A and Bmpr2a-A).** 
Yeast cells co-transformed with bait and prey plasmids that survived on TDO and QDO plate indicate weak and strong positive interactions, respectively. The pBT3-SUC-Gsdf-A + pPR3-N was used for detection of self-activation, while the pBT3-SUC-Gsdf-A + POST-Nubal was used for functional verification of the ubiquitin system. The pTSU2-APP + pPR3-N and pTSU2-APP + pNubG-Fe65 were used as the negative and positive control, respectively.

Through *in vitro* analyses, we demonstrated an interaction between Gsdf-A/B and Ncoa5, but we did not known whether there is a TGF-β type II receptor involved in this regulatory process by binding Gsdf. Intrigued by reviewer, we added an analysis of co-immunoprecipitation, in which the overexpression of these TGF-β type II receptors (Tgfbr2a-A, Tgfbr2b-A, and Bmpr2a-A) could not affect Ncoa5 recruitment by Gsdf-A. Thus, we think that these TGF-β type II receptors (Tgfbr2a-A, Tgfbr2b-A, and Bmpr2a-A) may be not involved in the interaction between Gsdf and Ncoa5 (Figure R2)

**Figure R2 Co-IP of Gsdf-A-Flag and Ncoa5-Myc with Tgfbr2a-HA, Tgfbr2b-HA, and Bmpr2a-HA in EPC cells transfected with the indicated plasmids.**

Anti-Flag Ab was used for Co-IP.

2) Usually, the male and female pathway genes display obvious sexually dimorphic expression. If both Ncoa5 and Rora are involved in sex differentiation, why there is no difference in the expression of ncoa5 and rora between females and males in the Figure 7A. Further, in Figure 7B, overexpression of gsdf led to downregulation of roar, why there was no difference in the expression of rora between females and males in the Figure 7A?

**Response:** Thanks for your comments. Rora (retinoic acid-related orphan receptor-alpha) is a member of hormone nuclear receptor superfamily. Rora can active expressions of several genes by binding to ROR response elements (ROREs) upstream of these genes. Previous studies have revealed that estrogen receptor (ER) and NCOA5 collaborate in the up-regulation of RORA by estradiol (Odawara *et al.*, 2009; Sarachana and Hu, 2013). Thus, overexpression of *gsdf* downregulates *cyp19a1a* and suppresses the production of estradiol, which may repress the activation of Rora by estradiol and ER/Ncoa5, leading to downregulation of *roar* as shown in the original Figure 7B.

In original Figure 7A (revised Figure 8A), the expression of *rora* has no difference between females and males before 45 dph but display female-biased expression at 60 dph. Thus, we have added the data of 60 dph in the revised Figure 8A and revised this paragraph, which is consistent with the *in vitro* results in the revised Figure 8C and 8D (original Figure 7B and 7C) (line 423-426 in the revised manuscript).

Ncoa5 (nuclear receptor coactivator 5) is a coregulator for nuclear receptors and has both coactivator and co repressor functions. It also can form complex with ER and enhance ER transcription in the presence of estradiol (Sauvé *et al.*, 2001). Ncoa5 exercise has ubiquitous expression without tissue-specificity. Also, Ncoa5 display no expression difference between females and males in gonads at early developmental stages (revised Figure 8A), which is consistent with the *in vitro* results in the revised Figure 8C and 8D (line 421-423 in the revised manuscript).

**Revised Fig 8. Gsdf inhibits *cyp19a1a* transcription via competitive interaction with Ncoa5.**

**(A)** Gene expression detected by qPCR in gonads during different developmental stages. MSM+, wild type males with MSM; MSM−, wild type females without MSM; **(B)** FISH analysis of the *rora* (Pink) and *ncoa5* (Red) mRNA, and immunofluorescence analysis of the Gsdf protein (Green) in mature testis. Arrowhead indicates the somatic cells with expression of *rora*, *ncoa5*, and Gsdf. Scale bars: 25 µm. **(C-D)** Gene expression detected by qPCR after overexpression of Gsdf-A **(C)** and Gsdf-B **(D)**. The asterisks indicate the significant differences (**P*<0.05, ***P*<0.01).

3) An important finding of this work is that in females Ncoa5/Rora induced activation of cyp19a1a independent of Foxl2/Sf1-cyp19a1a pathway. We all know that mutation of Foxl2 lead to female to male sex reversal. The specific function of Ncoa5 in fish and whether it is involved in fish sex differentiation has not been explored. For this reviewer, it is hard to believe Ncoa5/Rora-cyp19a1a and Foxl2/Sf1-cyp19a1a pathway is independent in female sex differentiation.

**Response:** Thanks for your comments and concerns. *In vitro* analyses reveal that Rora interacts with Ncoa5 to enhance *cyp19a1a* transcription. However, we supposed that the pathway of Ncoa5/Rora induced *cyp19a1a* activation is not independent of Foxl2/Sf1-*cyp19a1a* pathway. We have added the discussion in the revised manuscript: “*As we known, Foxl2/Sf1-cyp19a1a pathway is an important pathway of female sex differentiation, and knockout of foxl2 results in female to male sex reversal in fish(**Gan et al., 2021**;* *Yang et al., 2017**). We suppose that the pathway of Rora/Ncoa5 induced cyp19a1a activation is not independent of Foxl2/Sf1-cyp19a1a pathway. For instance, the cyp19a1a expression change induced by Foxl2/Sf1 can affect estradiol synthesis, which may lead to expression changes of er and rora in the presence of Ncoa5 and then affect cyp19a1a transcription via Rora/Ncoa5-cyp19a1a pathway (**Sarachana and Hu, 2013**;* *Sauvé et al., 2001**). Thus, these two pathways of cyp19a1a regulation may interact with each other.*” (line 475-482 in the revised manuscript)

4) In Figure 2D and 2E, the authors demonstrated that Dmrt1 and Sf1 activate gsdf expression through binding to their potential binding sites in the gsdf promoter, it is better to determine which site is important for gsdf activation. It is also better to demonstrate that Dmrt1 and Sf1 are co-expressed in gsdf expressing cells.

**Response:** Thanks for your suggestions. To reveal which Dmrt1-binding site is important for *gsdf* activation, we have performed promoter mutation analysis: “*Mutation of the Dmrt1-binding site 1 or 3 on gsdf-A potential promoter led to a decrease in both Dmrt1-A-induced and Dmrt1-B-induced transcriptional activation of gsdf-A. Meanwhile, Mutation of the Dmrt1-binding site 1 or 3 on gsdf-B potential promoter resulted in a decrease in both Dmrt1-A-induced and Dmrt1-B-induced transcriptional activation of gsdf-B. However, mutation of Dmrt1-binding site 2 on* *gsdf-A and gsdf-B did not significantly affect Dmrt1-A/Dmrt1-B-induced transcriptional activation (Fig 3C and 3D). Thus, Dmrt1-binding site 1 and 3 of both gsdf-A and gsdf-B were important for gsdf activation.*” (line 221-229 in the revised manuscript)

Besides, we have added co-localization *dmrt1*, *sf1*, and Gsdf in the revised manuscript: “*Fluorescence in situ hybridization (FISH) analysis showed that dmrt1 and sf1 mRNA were co-expressed in some Gsdf positive somatic cells in testis (Fig 3G).*” (line 233-235 in the revised manuscript)

**Revised Fig 3. Dmrt1 regulates *gsdf* transcription in the presence of Sf-1.**

**(A)** Putative Dmrt1 and Sf1 *cis*-regulatory elements in the potential promoters of *gsdf-A* and *gsdf-B*. **(B)** In the presence of Sf1 (Sf1-A 25 ng + Sf1-B 25 ng), Dmrt1-A/Dmrt1-B overexpression activates the promoter activity of *gsdf-A*/*gsdf-B* in a dosage-dependent manner (50, 100, 150 ng) in CAB cells. For *gsdf-A* potential promoter, the transcriptional regulation ability of Dmrt1-A was significantly stronger than that of Dmrt1-B. For *gsdf-B* potential promoter, the transcriptional regulation ability of Dmrt1-B was significantly stronger than that of Dmrt1-A. The asterisks indicate the significant differences (**P*<0.05, ***P*<0.01). **(C-D)** 5′-mutation mapping of Dmrt1-binding region on the *gsdf-A* **(C)** and *gsdf-B* **(D)** potential promoter. Blue boxes indicate predicted Dmrt1-binding sites. Red ‘×’ indicates the mutated binding site. The asterisks indicate the significant differences (**P*<0.05, ***P*<0.01, ****P*<0.001, *****P*<0.0001, ******P*<0.00001) , Different letters represent statistical differences (*P*<0.01). **(E-F)** qPCR of *dmrt1-A*/*dmrt1-B* transcripts in adult organs **(E)** and male gonads at different developmental stages **(F)**. The highest expression level in each qPCR analysis was used as control and defined as 1. **(G)** FISH analysis of the *dmrt1*(Red) and *sf-1* (Pink) mRNA, and immunofluorescence analysis of the Gsdf protein (Green) in mature testis. Arrowhead indicates the somatic cells with expression of *sf-1*, *dmrt1*, and Gsdf. Scale bars: 25 µm.

Miner concerns:
Line 172：“Expression plasmids of Sf1-A/Sf1-B, Dmrt1-A, Dmrt1-B, and an empty expression plasmid were used as controls” should be “Expression plasmids of Sf1-A/Sf1-B, Dmrt1-A, Dmrt1-B were constructed, and an empty expression plasmid was used as controls”.

**Response:** We have changed “Expression plasmids of Sf1-A/Sf1-B, Dmrt1-A, Dmrt1-B, and an empty expression plasmid were used as controls” into “Expression plasmids of Sf1-A/Sf1-B, Dmrt1-A, Dmrt1-B were constructed, and an empty expression plasmid was used as control” (line 214-215 in the revised manuscript).

Line 555: “mediaand” should be “medium”.

**Response:** We have corrected “mediaand” to “medium and” (line 660 in the revised manuscript).

Line 556: “luc” should be “luciferase reporter gene”.

**Response:** We have changed “luc” into “luciferase reporter gene” (line 662).

Line 633: A repeated copy of “under the accession number of” should be deleted.

**Response:** We are sorry about this careless mistake, and we have deleted the repeated copy of “under the accession number of”.

Line 904: “exam” should be “examine”.

**Response:** We have changed “exam” into “examine” (line 1033).

Line 908: “indicates” should be “indicate”.

**Response:** We have changed “indicates” into “indicate” (line 1038).

Line 921: “transcriptions” should be “transcripts” or “mRNA expression”, and the same for line 934.

**Response:** According to your advice, we have changed “transcriptions” into “transcripts”.

Line 958: The letter “e” in the Figure legend should be “(E)”.

**Response:** Thanks for pointing out this mistake. We have changed “e” into “(E)”.

Line 980: “activates the promoter activity of gsdf-A/gsdf-B”, the promoter described here should be the “cyp19a1a promoter”.

**Response:** Thanks you for pointing out this mistake. We have changed “activates the promoter activity of gsdf-A/gsdf-B” into “activates *cyp19a1a* promoter”.

Line 1071：“noca5” should be “ncoa5”.

**Response:** We have changed “noca5” into “ncoa5” (line 1274).

**Reviewer #3:**

This is an interesting and well-conducted study showing the role of a member of the transforming growth factor beta (TGF-ß) signaling pathway, gonadal somatic cell-derived factor (gsdf) to be involved in male differentiation in the hexaploid gibel carp (Carassius gibelio). This species was used as a system to investigate the mechanisms that duplicate genes use in a developmental process such as sex differentiation. Authors demonstrate the presence of two homeologues of gsdf, termed gsdf-A and gsdf-B that cooperatively regulate male sex differentiation by interacting with the nuclear co-activator factor 5 (Ncoa5) to suppress gonadal aromatase (cyp19a1a) gene expression. In lower vertebrates, it is well established that aromatase activity is necessary for estrogen production and female development. The study is very complete and includes the molecular characterization of gsdf-A and gsdf-B genes, expression during gonad development, transcriptional regulation, and functional analysis by wisely combining induced gynogenesis using males with or without a male determinant contained in supernumerary microchromosomes and loss-of-function analysis by the production of single or double mutants by means of CRISPR technology. Authors convincingly show that disruption of either gsdf-A or gsdf-B results in partial sex reversal while disruption of both homeologs results in complete male-to-female sex reversal. Furthermore, male-to-female sex reversal in double mutants is due to stimulation of cyp19a1a, the effects of which can be inhibited by treatment with a non-steroidal aromatase inhibitor. Authors identify Ncoa5 as interaction partner of Gsdf and show that gsdf binding of Ncoa5 prevents the latter to interact with Ncoa to upregulate cyp19a1 transcription. The study nicely shows how both forms of gsdf cooperate towards male sex differentiation. I have some comments that may help to improve the ms.

**Response:** Thank you very much for your positive comments.

L27 and Throughout the ms. The term “homeologous”, used in polyploidy plant research, may not be familiar with some readers, who may confuse it with “homologous”. Please explain that homologous genes resulting from allopolyploidy are commonly referred to as “homoeologs”. Here you use the term “homeologs”, which I am not sure how common it is. All this may create confusion. Please provide a clear definition of the term and its possible variants right from the start.

**Response:** Thanks for your valuable suggestion. We have added the definition of hemeologs (also homoeologs) in the Introduction part as follows: “*Polyploidy may* *inherit additional set/sets of chromosomes from the same species (autopolyploidy) or from interspecific hybridization (allopolyploidy)(**Otto, 2007**). Homologous chromosomes (and the genes they carry) resulting from allopolyploidy are commonly referred to as* *homeologs (also homoeologs)(**Soltis et al., 2015**)*.” (line 89-92 in the revised manuscript)

L. 91-93. “a variant of gynogenesis”. This needs to be better explained to readers. Please indicate ploidy level of each progenitor in the initial cross. Also explain whether there is a dose-dependent relationship between the microchromosomes and the percent of males.

**Response:** According to your suggestions, we have explained “a variant of gynogenesis”, indicated ploidy level of paternal individuals, and also explained whether there is a dose-dependent relationship between the microchromosomes and the percent of males: “*In our previous studies, we identified a genetic male-specific marker (MSM) in gibel carp (**Wang et al., 2009**) and revealed that* *male-specific supernumerary microchromosomes are closely associated with the occurrence of genotypic males in a dose-dependent relationship (**Ding et al., 2021**;* *Li et al., 2016**). When a female gibel carp is mated with a male from other species, a typical gynogenesis is initiated that all the offspring have the same genetic information as the maternal individual. When a female gibel carp is mated with a genotypic amphitriploid male, a variant of gynogenesis is initiated, during which the sperm nuclei are also extruded but some supernumerary microchromosomes of sperm nuclei occasionally leak into the unreduced eggs. This variant of gynogenesis can accumulate microchromosomes, generate males, and create genetic diversity in the offspring*.” (line 123-133 in the revised manuscript)

L109. I am not sure if it would be more appropriate to speak about “sex determination” rather than to “sex differentiation”. Duplicates of gsdf has been implicated in sex determination. In fact, in lines 101-102 authors write “Members of the transforming growth factor-β (TGF-β) signaling pathway have been identified as being vastly involved in sex determination”.

**Response:** Thanks for your comments. Sex determination and sex differentiation are two processes of sexual development. Sex determination determines whether the bipotential primordium develops into an ovary or testis by genetic, environment factors, or both. Sex differentiation takes place after sex determination and leads to the actual differentiation and development of gonads.

We have revised “*Members of the transforming growth factor-β (TGF-β) signaling pathway have been identified as being vastly involved in sex determination” into “Members of the transforming growth factor-β (TGF-β) signaling pathway have been identified as being vastly involved in sex determination and differentiation*” (line 138-139 in the revised manuscript).

In gibel carp, *gsdf* genes (*gsdf-A* and *gsdf-B*) have no difference of DNA sequence between sexes, indicating that *gsdf* genes are sex differentiation genes and not sex-determining genes. Thus, we though it is more appropriate to speak about “sex differentiation” rather than to “sex determination” here.

L151. Gsdf-A expression during sex differentiation peaked at 250 dph while the expression of Gsdf-B peaked at 120 dph. However, gonadal morphological differentiation between males and females occurs around 40 dpf. How then it is possible that Gsdf-A and B are drivers of sex determination/differentiation?

**Response:** Thanks for your comments. For relative qPCR analysis, *β-actin* was used as internal reference and relative expression level of target gene was calculated with 2^-ΔΔCT^ method. In the original Figure 2B, the expression of *gsdf-A* at 250 dph was used as control and defined as 1. Since the expression level of *gsdf-A* at 250 dph was so high that the basal relative expressions of *gsdf-A* and *gsdf-B* at 17-30 dph appeared to be low. When compared with the *gsdf* expressions in female gonads, the relative expressions of *gsdf* at 18-30 dph appeared to be significantly higher (revised Figure 8A). Thus, *gsdf-A* and *gsdf-B* may drive male differentiation in gibel carp.

**Revised Fig 8A. *gsdf* expression detected by qPCR in gonads during different developmental stages.**

MSM+, wild type males with MSM; MSM−, wild type females without MSM. The expression level in male gonads at 60 dph was used as control and defined as 1.

L166-168. Consider rephrasing the sentence to clearly indicate that the identified upstream sequences of gsdf-A and gsdf-B were considered the potential promoter sequences; otherwise it seems that the binding sites per se are the promoter regions.

**Response:** We apologize for the confusing descriptions and we have rephrased this sentence as follows: “*By in silico analysis, three predicted Dmrt1-binding sites and two predicted Sf1(also named as Nr5a1)-binding sites (**Murphy et al., 2015**) were identified in the upstream sequences of both gsdf-A (from -2080 to +50) and gsdf-B (from -2150 to +50), which were defined as potential promoter of gsdf-A (2130 bp) and gsdf-B (2200 bp), respectively (Fig 3A).*” (line 207-211 in the revised manuscript)

L191. Indicate how did you ensure that the female gibel carp was devoid of MSM.

**Response:** Thanks for your suggestion. To determine whether the female gibel carp contained MSM, we performed PCR analysis using the specific primers for MSM. And we have added a paragraph of “DNA extraction and PCR detection of MSM” in the Materials and Methods section: “***DNA extraction and PCR detection of MSM:***

*A small piece of fin was used to extract genomic DNA for each sampled fish, using DNA extraction kit (Promega) according to the manufacturer’s instructions. The MSM was detected by PCR using the primer pair Cg-MSM-F and Cg-MSM-R(**Li et al., 2016**). PCR analysis was performed as previously described (**Zhao et al., 2021**).*” (line 615-619 in the revised manuscript)

L263. Delete “obviously”.

**Response:** We have deleted “obviously” in the revised manuscript.

L276. Add “nonsteroidal” before “aromatase inhibitor”.

**Response:** Thanks for your suggestions and we have added “nonsteroidal” before “aromatase inhibitor” (line 335 in the revised manuscript).

L277. Add “enzyme activity” after “Cyp19a1a”.

**Response:** According to your advice, we have added “enzyme activity” after “Cyp19a1a” (line 336 and 346 in the revised manuscript).

L279. Explain why there were approximately 15% of the fish in which male-to-female sex reversal could not be prevented.

**Response:** Thanks for your comments and we have added the explanation in the revised manuscript: “*As the period before 15 dph was key developmental stages of sex determination/differentiation, letrozole treatment after 15 dph might be the reason that 15.6% gsdf-A/gsdf-B double mutants still developed into phenotypic females.*” (line 339-342 in the revised manuscript)

L282. Upregulation of foxl2b in the testis of gsdf-A/gsdf-B double mutants (MSM+) subjected to letrozole treatment comes as a bit of surprise since usually foxl2a and Cyp19a1a expression goes hand in hand due to the regulatory positive feedback loop involving these two genes. Please clarify.

**Response:** Intrigued by your comments, we have added the *cyp19a1a* expression in the revised Figure 5G. As you said, *foxl2* and *cyp19a1a* expression goes hand in hand: “*And gene expression patterns of these letrozole treated testes were similar to that in WT males (MSM+), except for cyp19a1a and foxl2b (Fig 5G). Upregulation of cyp19a1a and its active transcriptional factor foxl2b (**Gan et al., 2021**;* *Wang et al., 2007**)in the testis of gsdf-A/gsdf-B double mutants (MSM+) subjected to letrozole treatment may have been caused by inhibition of Cyp19a1a enzyme activity.*” (line 342-346 in the revised manuscript)

**Revised Fig 5G. Gene expression of *gsdf-A*/*gsdf-B* double mutant individuals (*gsdf-A*^Δ4,+9/Δ4/Δ7^ + *gsdf-B* ^chimeric mutations^, MSM+), *gsdf-A*/*gsdf-B* double mutant individuals (*gsdf-A*^Δ4,+9/Δ4/Δ7^ + *gsdf-B* ^chimeric mutations^, MSM+) with aromatase inhibitor (Letrozole) treatment, and wild type individuals at 100 dph.**

Different letters represent statistical differences (*P*<0.05). The highest expression level in each qPCR analysis was used as control and defined as 1.

L317. How can you be sure that Ncoa5-A had no role in sex differentiation

Response: Thanks for your comments. We think that Ncoa5-A may also be involved in sex differentiation, as the protein sequences between Ncoa5-A and Ncoa5-B was highly conserved (identity = 91.53%). The genes involved in *gsdf*-*cyp19a1a* pathway all have many duplicates, such as *ncoa5-A*, *ncoa5-B*, *roraα-A*, *roraα-B*, *roraβ-A*, *roraβ-B*, *cyp19a1a-A*, and *cyp19a1a-B*. To make the interaction results more simple and clear, we only used these genes from subgenome B (*ncoa5-B*, *roraβ-B*, and *cyp19a1a-B*) for subsequent *in vitro* experiments.

L394. It comes as a bit of surprise that no further explanation of the different functions of gsdf-A and gsdf-B are discussed. This is relevant to the study but these functions are not properly explained. This is relevant in view of the overall aim of the paper. It should be stated whether the contribution of each homeologue is similar, if there is any indication that one has more relevance than the other in male differentiation.

**Response:** Thanks for your comments. We are sorry about the arbitrary conclusion. Knockout *gsdf-A* or *gsdf-B* displayed similar sex-reversal rates (80.6% and 85.3% sex reversal rates in *gsdf-A* mutant lines; 83.6% and 80.0% sex reversal rates in *gsdf-B* mutant lines), indicating the contribution of each homeolog is similar in male differentiation. So we have revised this paragraph as follows: “*Here, we found expression divergence between gsdf-A and gsdf-B (Fig 2A and 2B), but knockout gsdf-A or gsdf-B displayed similar sex-reversal rates (S5 Fig), indicating the contribution of each homeolog is similar in male differentiation. In addition, disruption of gsdf-A or gsdf-B triggers highly compensatory expression of gsdf-B or gsdf-A* *during the critical period of sex differentiation (Fig 5E), and missing either gsdf-A or gsdf-B does not give complete sex reversal but missing both does, suggesting that gsdf-A and gsdf-B cooperatively regulate male differentiation in gibel carp.*” (line 454-461 in the revised manuscript)

L421. “Thus, dysfunction of gsdf triggers upregulation of cyp19a1a and subsequently leads to the inhibition of dmrt1 and activation of foxl2”. I would rephrase a bit this sentence because in this study the whole chain of events is not shown. So I suggest indicating that, as shown in previous studies, on one hand upregulation of cyp19a1a would lead to dmrt1 inhibition while on the other it would upregulate foxl2.

**Response:** Thanks for your suggestion. We have rewritten this sentence as follows: “*As shown in previous studies, on one hand upregulation of cyp19a1a would lead to dmrt1 inhibition while on the other it would upregulate foxl2.*” (line 494-496 in the revised manuscript)

References for response letter:

Chen Y, Hong WS, Wang Q, Chen SX. Cloning and pattern of *gsdf* mRNA during gonadal development in the protogynous *Epinephelus akaara*. Anim Reprod Sci. 2016;165:46-55. https://doi.org/10.1016/j.anireprosci.2015.12.004 PMID: 26718719

Derynck R, Budi EH. Specificity, versatility, and control of TGF-beta family signaling. Sci Signal. 2019;12(570):eaav5183. https://doi.org/10.1126/scisignal.aav5183 PMID: 30808818

Ding M, Li XY, Zhu ZX, Chen JH, Lu M, Shi Q, et al. Genomic anatomy of male-specific microchromosomes in a gynogenetic fish. PLoS Genet. 2021; 17(9):e1009760. https://doi.org/10.1371/journal.pgen.1009760 PMID: 34491994

Gan RH, Wang Y, Li Z, Yu ZX, Li XY, Tong JF, et al. Functional divergence of multiple duplicated foxl2 homeologs and alleles in a recurrent polyploid fish. Mol Biol Evol. 2021; 38(5):1995-2013. https://doi.org/10.1093/molbev/msab002 PMID: 33432361

Kaneko H, Ijiri S, Kobayashi T, Izumi H, Kuramochi Y, Wang DS, et al. Gonadal soma-derived factor (gsdf), a TGF-beta superfamily gene, induces testis differentiation in the teleost fish *Oreochromis niloticus*. Mol Cell Endocrinol. 2015; 415:87-99. https://doi.org/10.1016/j.mce.2015.08.008 PMID: 26265450

Li XY, Zhang QY, Li Z, Li Z, Zhang XJ, Wang D, et al. Extra microchromosomes play male determination role in polyploid gibel carp. Genetics. 2016; 203(3):1415-24. https://doi.org/10.1534/genetics.115.185843 PMID: 27017622

Murphy MW, Lee JK, Rojo S, Gearhart MD, Kurahashi K, Banerjee S, et al. An ancient protein-DNA interaction underlying metazoan sex determination. Nat Struct Mol Biol. 2015; 22(6):442-51. https://doi.org/10.1038/nsmb.3032 PMID: 26005864

Myosho T, Otake H, Masuyama H, Matsuda M, Kuroki Y, Fujiyama A, et al. Tracing the emergence of a novel sex-determining gene in medaka, *Oryzias luzonensis*. Genetics. 2012; 191(1):163-70. https://doi.org/10.1534/genetics.111.137497 PMID: 22367037

Odawara H, Iwasaki T, Horiguchi J, Rokutanda N, Hirooka K, et al. Activation of aromatase expression by retinoic acid receptor-related orphan receptor (ROR) alpha in breast cancer cells: identification of a novel ROR response element. J Biol Chem. 2009;284(26): 17711-17719. https://doi.org/10.1074/jbc.M109.009241 PMID: 19439415

Otto SP. The evolutionary consequences of polyploidy. Cell. 2007; 131(3):452-62. https://doi.org/10.1016/j.cell.2007.10.022 PMID: 17981114

Sarachana T, Hu VW. Differential recruitment of coregulators to the RORA promoter adds another layer of complexity to gene (dys) regulation by sex hormones in autism. Mol Autism. 2013; 4(1):39. https://doi.org/10.1186/2040-2392-4-39 PMID: 24119295

S Sauvé F, McBroom LDB, Gallant J, Moraitis AN, Labrie F, Giguère V. CIA, a novel estrogen receptor coactivator with a bifunctional nuclear receptor interacting determinant. Mol Cell Biol. 2001; 21(1):343-53. https://doi.org/10.1128/MCB.21.1.343-353.2001 PMID: 11113208

Shibata Y, Paul-Prasanth B, Suzuki A, Usami T, Nakamoto M, Matsuda M, et al. Expression of gonadal soma derived factor (GSDF) is spatially and temporally correlated with early testicular differentiation in medaka. Gene Expr Patterns. 2010;10(6):283-289. https://doi.org/10.1016/j.gep.2010.06.005 PMID: 20601164

Soltis PS, Marchant DB, Van de Peer Y, Soltis DE. Polyploidy and genome evolution in plants. Curr Opin Genet Dev. 2015; 35:119-25. https://doi.org/10.1016/j.gde.2015.11.003 PMID: 26656231

Wang DS, Kobayashi T, Zhou LY, PaulPrasanth B, Ijiri S, Sakai F, et al. Foxl2 up-regulates aromatase gene transcription in a female-specific manner by binding to the promoter as well as interacting with Ad4 binding protein/steroidogenic factor 1. Mol Endocrinol. 2007;21(3):712-25. https://doi.org/10.1210/me.2006-0248 PMID: 17192407

Wang D, Mao HL, Peng JX, Li XY, Zhou L, Gui JF. Discovery of a male-biased mutant family and identification of a male-specific SCAR marker in gynogenetic gibel carp *Carassius auratus* *gibelio*. Prog Nat Sci. 2009;19(11):1537-44. https://doi.org/10.1016/j.pnsc.2009.04.008

Yang YJ, Wang Y, Li Z, Zhou L, Gui JF. Sequential, divergent, and cooperative requirements of *foxl2a* and *foxl2b* in ovary development and maintenance of zebrafish. Genetics. 2017;205(4):1551-72. https://doi.org/10.1534/genetics.116.199133 PMID: 28193729

Zhang Y, Alexander PB, Wang XF. TGF-beta family signaling in the control of cell proliferation and survival. Cold Spring Harbor Perspect Biol. 2017;9(4):a022145. https://doi.org/10.1101/cshperspect.a022145 PMID: 27920038

Zhao X, Li Z, Ding M, Wang T, Wang MT, Miao C, et al. Genotypic males play an important role in the creation of genetic diversity in gynogenetic gibel carp. Front Genet. 2021; 12(875):691923. https://doi.org/10.3389/fgene.2021.691923 PMID: 34122529
